# Rare variants regulate expression of nearby individual genes in multiple tissues

**DOI:** 10.1371/journal.pgen.1009596

**Published:** 2021-06-01

**Authors:** Jiajin Li, Nahyun Kong, Buhm Han, Jae Hoon Sul

**Affiliations:** 1 Department of Human Genetics, David Geffen School of Medicine, University of California, Los Angeles, Los Angeles, California, United States of America; 2 Department of Biological Sciences, Korea Advanced Institute of Science and Technology, Daejeon, Republic of Korea; 3 Department of Medicine, Seoul National University College of Medicine, Seoul, Republic of Korea; 4 Department of Psychiatry and Biobehavioral Sciences, University of California, Los Angeles, Los Angeles, California, United States of America; Yale School of Medicine, UNITED STATES

## Abstract

The rapid decrease in sequencing cost has enabled genetic studies to discover rare variants associated with complex diseases and traits. Once this association is identified, the next step is to understand the genetic mechanism of rare variants on how the variants influence diseases. Similar to the hypothesis of common variants, rare variants may affect diseases by regulating gene expression, and recently, several studies have identified the effects of rare variants on gene expression using heritability and expression outlier analyses. However, identifying individual genes whose expression is regulated by rare variants has been challenging due to the relatively small sample size of expression quantitative trait loci studies and statistical approaches not optimized to detect the effects of rare variants. In this study, we analyze whole-genome sequencing and RNA-seq data of 681 European individuals collected for the Genotype-Tissue Expression (GTEx) project (v8) to identify individual genes in 49 human tissues whose expression is regulated by rare variants. To improve statistical power, we develop an approach based on a likelihood ratio test that combines effects of multiple rare variants in a nonlinear manner and has higher power than previous approaches. Using GTEx data, we identify many genes regulated by rare variants, and some of them are only regulated by rare variants and not by common variants. We also find that genes regulated by rare variants are enriched for expression outliers and disease-causing genes. These results suggest the regulatory effects of rare variants, which would be important in interpreting associations of rare variants with complex traits.

## Author summary

It has been shown that rare variants may affect many diseases including both rare and common diseases with the advent of next-generation sequencing technology. An important question is how rare variants affect diseases or traits, especially whether or how they regulate gene expression as they may affect diseases through gene regulation. However, it is challenging to identify the regulatory effects of rare variants because it often requires large sample sizes and the existing statistical approaches are not optimized for it. Here, we develop a novel method, LRT-q, based on a likelihood ratio test that aggregates the effects of multiple rare variants nonlinearly to achieve higher statistical power than previous rare variant association methods. We apply LRT-q to the latest GTEx v8 dataset and identify regulatory effect of rare variants on individual genes. We also observe that genes regulated by rare variants are likely to be disease-causing genes. These results demonstrate the functional effects of rare variants, especially on gene expression, which provides important biological insights in understanding the genetic mechanism of rare variants in complex traits and diseases.

## Introduction

Over the past decade, genome-wide association studies (GWAS) have successfully discovered numerous associations between common genetic variants and human complex diseases and traits[1,2]. These studies also found that those common variants typically have small effects and explain a small fraction of heritability[3,4]. Motivated by this finding, many sequencing studies have attempted to identify rare variants associated with complex traits[5,6]. It is hypothesized that rare variants may have larger effect sizes than common variants due to purifying selection and may explain some of the missing heritability[7,8]. Candidate-gene and large-scale sequencing studies have indeed found associations of rare variants with complex diseases and traits[9–11].

An important question after finding the associations of rare variants is to understand their genetic mechanism on how they influence diseases. GWAS have found that common variants associated with diseases are mostly present in non-coding regions of the genome, suggesting that they might affect traits by regulating the expression of nearby genes as recent expression quantitative trait loci (eQTL) studies have identified many common variants with regulatory effects[12,13]. However, the effect of rare variants on gene expression remains mostly obscure, although there have been recent developments in this work. For example, Li et al. discovered that rare variants might result in outlier patterns of over or under expression across multiple human tissues[14] while Zhao et al. found an excess of rare variants was significantly associated with extreme gene expression in human peripheral blood[15]. Hernandez et al. also reported that ultrarare variants make a significant contribution to the heritability of gene expression[16]. These results hint at the possible functional effect of rare variants.

To discover the functional effect of genetic variants, many eQTL studies are interested in identifying genes whose expression levels are influenced by genetic variants (called “eGenes”). The aforementioned studies for rare variants mostly focused on the overall contribution of rare variants to gene expression but did not find individual genes whose expression is associated with rare variants. We call these genes “RV eGenes,” and there are two major challenges in finding RV eGenes. The first is relatively small sample sizes of eQTL studies that collected whole-genome sequencing (WGS) as well as RNA-seq data. WGS data instead of whole-exome sequencing data are necessary to discover RV eGenes as many variants regulating gene expression may be present in non-coding regions of the genome. The second challenge is the statistical approach to detect eGenes. While there have been several methods developed to identify common variant eGenes (CV eGenes)[17,18], these methods utilize a single marker test that tests each SNP, which yields low statistical power for rare variants. To increase power to detect association of rare variants, many collapsing approaches that combine the effect of multiple rare variants have been proposed[19,20], but as we will discuss later, they are not optimized to find RV eGenes.

In this paper, we develop a powerful approach called LRT-q to detect RV eGenes and apply this method to WGS and multi-tissue RNA-seq data collected from 681 European individuals in the Genotype-Tissue Expression (GTEx) project (v8). LRT-q incorporates functional annotations of rare variants, observational genotype data, and quantitative phenotype data to identify a group of potential causal rare variants influencing the expression of a nearby gene by aggregating statistics of rare variants in a nonlinear manner. We show using extensive simulations that LRT-q outperforms previous methods for rare variant association testing such as SKAT-O[21] and variable threshold[22]. We also find that LRT-q detects more RV eGenes than previous methods in the GTEx data across all tissues. We investigate the characteristics of those RV eGenes and discover a few important biological insights such as higher tissue specificity of RV eGenes compared to CV eGenes and enrichment of RV eGenes in disease-associated genes. We provide an open-source R package implementing the proposed method, LRTq.

### Overview of LRT-q

An association test between a single rare variant and expression of a gene is likely to result in low statistical power because the power decreases as allele frequency of a variant decreases. To overcome this challenge, many statistical approaches have been developed to aggregate rare variants in a genetic region, like a gene, and to test their cumulative effects on a phenotype. The underlying rationale is that a gene can be regulated by multiple rare variants and thus a larger effect can be observed by grouping them, contributing to increased power. The methods for rare variant testing include burden tests like variable threshold (VT)[22], variance component tests like sequence kernel association test (SKAT)[23], and combined tests like SKAT-O[21,24].

These methods, however, may not be optimal in detecting the effects of rare variants because of the following two reasons. First, they do not attempt to prioritize likely causal variants. As the rare variant methods combine multiple variants, it is important to remove the effects of non-causal variants. Previous methods mostly rely on functional information of variants to prioritize variants[20,25] such as minor allele frequency (MAF) and Combined Annotation Dependent Depletion (CADD) scores[26] as it has been hypothesized that rarer variants may have larger effects than more common variants. However, we may be able to prioritize potential causal variants more accurately by using both functional information and genotype data where the latter may provide additional information on the causal statuses of variants. For example, for gene expression data, individuals with causal rare variants may have significantly different expression patterns from other individuals. The other reason why previous methods may not be optimal is that many of the burden tests combine statistics of multiple variants linearly (e.g. a weighted sum of z-scores). However, it may be desirable to combine the statistics in a nonlinear manner to detect more associations as we show in our results.

To overcome these limitations, we propose a likelihood ratio test for quantitative traits (LRT-q) for detecting rare variants associated with gene expression. This method is an extension of the original LRT[27] that was designed for identifying associations between rare variants and disease status (dichotomous traits). There are two underlying models in LRT-q; 1) the null model that assumes no causal variants among all rare variants, and 2) the alternative model that assumes at least one causal variant. LRT-q calculates a likelihood ratio statistic between the two models and also a p-value using a permutation test. LRT-q calculates the statistic using functional information and observational genotype data that allows LRT-q to prioritize potential causal rare variants. Besides, LRT-q aggregates statistics of multiple rare variants nonlinearly to boost statistical power (see Materials and Methods). Assuming that individuals carrying a rare allele of a variant have different gene expression patterns from those carrying a different allele, we calculate a statistic measuring this difference for each rare variant near a gene. We then combine these statistics from multiple rare variants nonlinearly and generate an aggregated statistic for the gene. LRT-q considers both positive and negative effect sizes of genetic variants on gene expression, and it is very efficient using an adaptive permutation test.

### Verification and comparison

#### False positive rate of LRT-q

To measure the performance of LRT-q, we first measure the false positive rate using simulated data under the null hypothesis of no causal variants (see Materials and Methods). Each simulation has 1,000 individuals and 33 rare variants on average, and we test several other rare variant association methods such as CMC[28], WSS[29], Burden, VT[22], ACAT-V, ACAT-O[20], and SKAT-O[21] in addition to LRT-q. Here, ACAT-O is an omnibus test constructed by combining p-values of VT, ACAT-V, and SKAT-O. Results show that all methods have well-controlled false positive rates across different significance thresholds such as *α* = 0.05, 0.01, 0.001, and 0.0001 (Table 1).

**Table 1:** False positive rate of eight rare variant test methods in simulation.

*α*	CMC	WSS	Burden	VT	SKAT-O	ACAT-V	ACAT-O	LRT-q
0.05	0.04935	0.04765	0.04904	0.05134	0.05036	0.04971	0.04916	0.04975
0.01	0.00958	0.00929	0.00988	0.01021	0.01055	0.00973	0.01006	0.01015
0.001	1.17×10^−3^	1.04×10^−3^	1.11×10^−3^	0.99×10^−3^	1.32×10^−3^	1.00×10^−3^	1.00×10^−3^	0.89×10^−3^
0.0001	1.00×10^−4^	1.10×10^−4^	1.20×10^−4^	1.10×10^−4^	1.10×10^−4^	1.20×10^−4^	0.50×10^−4^	0.90×10^−4^

#### Power of LRT-q

Next, we perform power simulation under the alternative hypothesis that there is at least one causal rare variant using several combinations of effect sizes and causal ratio where causal ratio defines the percentage of causal variants among all rare variants (see Materials and Methods). In simulations, half of the causal variants have positive effect sizes, and the rest of causal variants have negative effect sizes as rare variants might increase or decrease expression levels. Additionally, as only a few rare variants might be causal, only 3% to 10% of rare variants are causal in the simulation data. Regarding effect sizes, variants with lower allele frequency have larger effect sizes, which is the assumption often made in simulating the effect of rare variants, and we simulate several different maximum effect sizes of rare variants (from 0.99 to 4.95). For each combination of effect size and causal ratio, we generate 10,000 datasets containing 1,000 subjects. Similar to the false positive rate simulation, we test eight methods, and the power is measured at *α* = 0.05.

**Fig 1:**
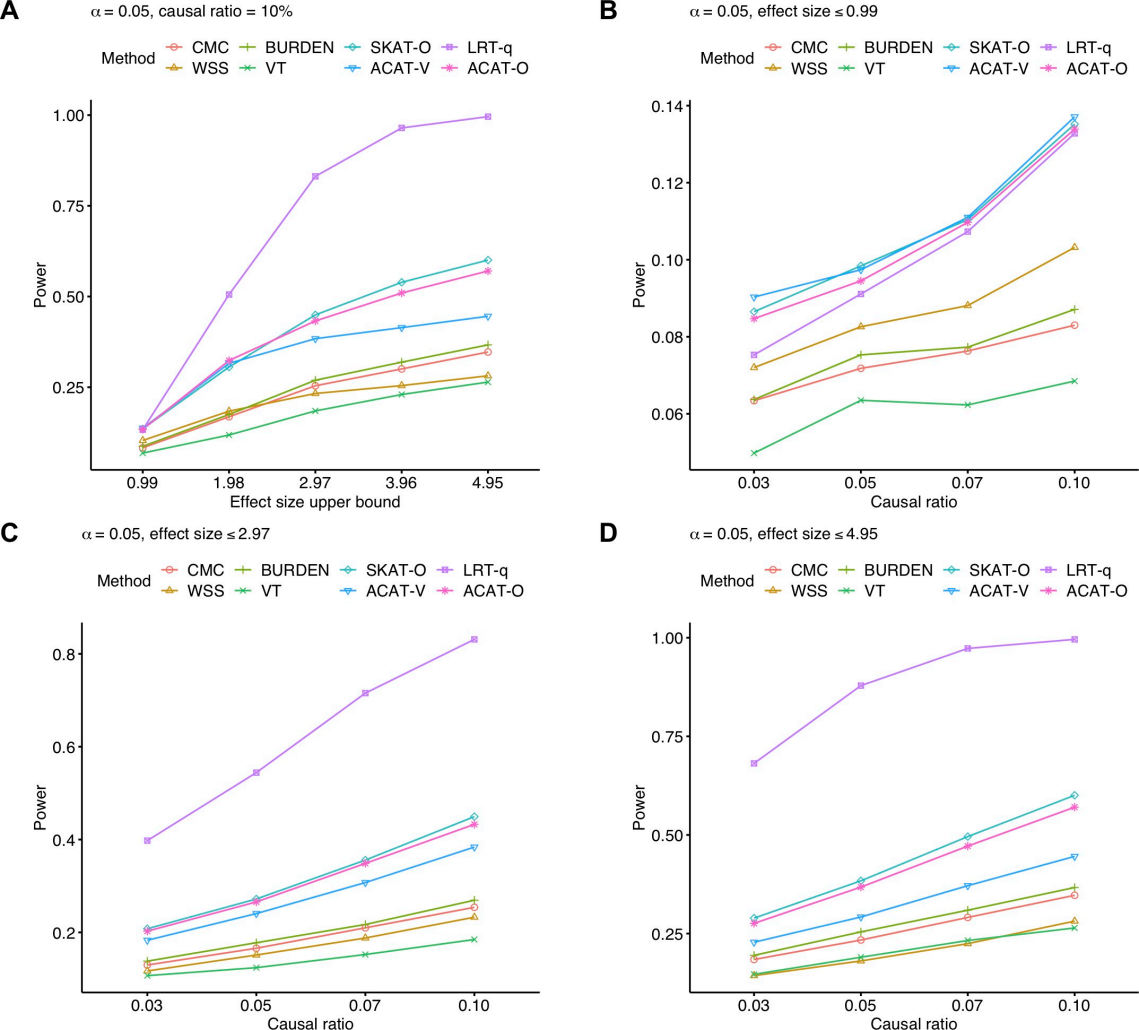
Power comparison between LRT-q and seven existing methods on simulated data. A. for different effect sizes and fixed causal ratio (10%), and for fixed effect sizes (B. ≤ 0.99, C. ≤ 2.97, D. ≤ 4.95) and various causal ratios. Significance level α = 0.05.

Results show higher power of LRT-q over other methods across a variety of simulation settings (Fig 1). Especially, we observe that as the effect size or the proportion of causal variants increases, LRT-q becomes more powerful than other approaches. When 10% of rare variants are causal, LRT-q has the highest statistical power if the maximum effect size is larger than or equal to 1.98. Its power is 147% to 224% as high as the power of SKAT-O, the second most powerful method. Furthermore, the power of LRT-q is slightly smaller or as large as the power of SKAT-O when the effect size of causal rare variants is very small (at most 0.99) (Fig 1A and 1B); in this case, all methods have very low power (<15%). When the effect size is larger, our method is considerably more powerful than SKAT-O (Fig 1C and 1D) where in these settings, the power of the proposed method is 141% to 238% as high as that of SKAT-O. These results demonstrate that prioritizing potential causal variants using the likelihood ratio test boosts statistical power to detect the effects of rare variants across various values of effect size and causal ratio.

Additionally, we perform simulations that add randomness to the effect sizes of causal rare variants by adding noises sampled from a normal distribution *N*(1,1). We also include simulations that contain much fewer rare variants (19.8 rare variants on average), which is about two-thirds of the number of rare variants in the original simulation (33.1). Note that it is difficult to further decrease the number of rare variants in the new simulations because the proportion of causal rare variants is assumed to be 3–10% and we need to ensure there is at least one causal rare variant. We still observe that LRT-q is much more powerful than other methods in these different settings (S1 Fig). For the convenience of parameter estimation, the LRT-q model assumes the equal variance of the expression levels of individuals with or without causal rare variants. To examine its robustness when the assumption is violated, we simulate the subjects that carry causal rare variants to have an explicitly different variance from those who do not. We find that our method is robust against the violation of this assumption and can still achieve higher power than other methods (S2 and S3 Figs). Therefore, this assumption simplifies the parameter estimation for the model but does not seem to influence its statistical power and reliability much.

One of the key reasons for the higher statistical power of LRT-q is its nonlinear decision boundary to detect significant associations. A decision boundary of an algorithm determines how it classifies each test (e.g. association test between rare variants and gene expression) into a significant or non-significant association where methods combining effect of rare variants linearly such as CMC have a linear decision boundary while LRT-q that applies nonlinear aggregation of rare variant effects have a nonlinear decision boundary. Using simulations to visualize decision boundaries of multiple rare variant methods (see Materials and Methods), we verify LRT-q has a clear nonlinear decision boundary that separates significant and non-significant associations accurately (S4A Fig). As the nonlinear decision boundary of LRT-q allows it to emphasize contributions from potential causal variants with large effects to its statistic more strongly than those from non-causal variants, LRT-q is more sensitive to causal effects and thus has higher power as demonstrated in the simulation studies above. Decision boundaries of other methods, however, are not as nonlinear as or as obvious as that of LRT-q because some of the significant associations detected by other methods overlap with non-significant associations (S4B–S4D Fig). In other words, the nonlinear decision boundary of LRT-q has higher accuracy in segregating rare variants with causal effects and those without causal effects, which improves statistical power.

### Applications

#### Identification of RV eGenes across 49 tissues in GTEx

To demonstrate the utility of our method in real eQTL data, we apply LRT-q to whole-genome sequencing (WGS) and RNA-seq data of 681 individuals with European ancestry from 49 tissues in the GTEx v8 dataset[30] to identify RV eGenes (see Materials and Methods for quality control and data processing); those are genes whose expression is regulated by nearby rare variants. We define rare variants as variants with MAF < 5% among individuals with WGS data, and we combine effects of rare variants present within 20K bp of a transcription start site (TSS) of each gene in each tissue. The GTEx study included common variants with MAF ≥ 1% in their eQTL analysis. This means that there may be some overlaps between rare variants in our analysis and common variants in the GTEx analysis as we used rare variants with MAF < 5%. Hence, we also analyzed rare variants with MAF < 1% to avoid this overlap. The GTEx study analyzed variants within 1 Mb from TSS while we use the 20 Kb window size. The main reason is that the number of rare variants within 1 Mb from TSS is considerably greater than that within 20 kb; we observe 50 times more rare variants within 1 Mb (median of 15,482) than 20 Kb (median of 311) as shown in S5 Fig. Including too many rare variants in a rare variant association test not only will greatly increase the computational cost but also is likely to decrease the power as more non-causal variants are included in the association test. Besides, previous studies[14,31] that analyzed the genetic effects of rare variants on gene expression also considered a smaller window size such as 10 Kb. In our analysis, we choose the window size that is twice as large as that of previous studies to include more variants with potential regulatory effects on gene expression. The sample size varies considerably depending on a tissue type (from 64 to 573) as only subsets of individuals provide RNA-seq data for certain tissues while we have WGS data for the 681 European individuals. To improve power to detect RV eGenes using LRT-q, we utilize a variety of weighting schemes such as assigning them uniform weights and prioritizing them by minor allele frequency (MAF), by their distances to TSS, and by their functional scores such as LINSIGHT[32] and CADD scores[33] as well as different combinations of them (see Materials and Methods). We then compare the performance of our method with that of other methods including ACAT-O, SKAT-O, and VT by applying the same weighting schemes to each method. It is important to note that CMC, Burden, and WSS are not included in this analysis because they have low power as demonstrated in the simulation study. ACAT-V is not under consideration because it uses an aggregated Cauchy association test as ACAT-O does but has lower power than ACAT-O as shown in simulation. We use a false discovery rate (FDR) of 5% to detect RV eGenes in each tissue.

We observe that different weighting schemes of rare variants yield very different numbers of RV eGenes and that our method detects more RV eGenes than other approaches across most of the weighting schemes. Using the whole blood (N = 546) as an example, LRT-q detects more RV eGenes than VT across all eight weighting strategies while we find more RV eGenes than ACAT-O and SKAT-O across four weighting schemes (S1 Table). Regarding the number of eGenes detected using different weighting schemes, the smallest number of RV eGenes LRT-q detects is 211 with TSS distance weighting while we observe about four times as many RV eGenes with a combined weight of MAF and CADD (885). These results show that consistent with the results of our power simulation, our method can detect more RV eGenes in real eQTL data than previous methods and that different weighting schemes could greatly influence the sensitivity of RV eGene detection.

**Fig 2:**
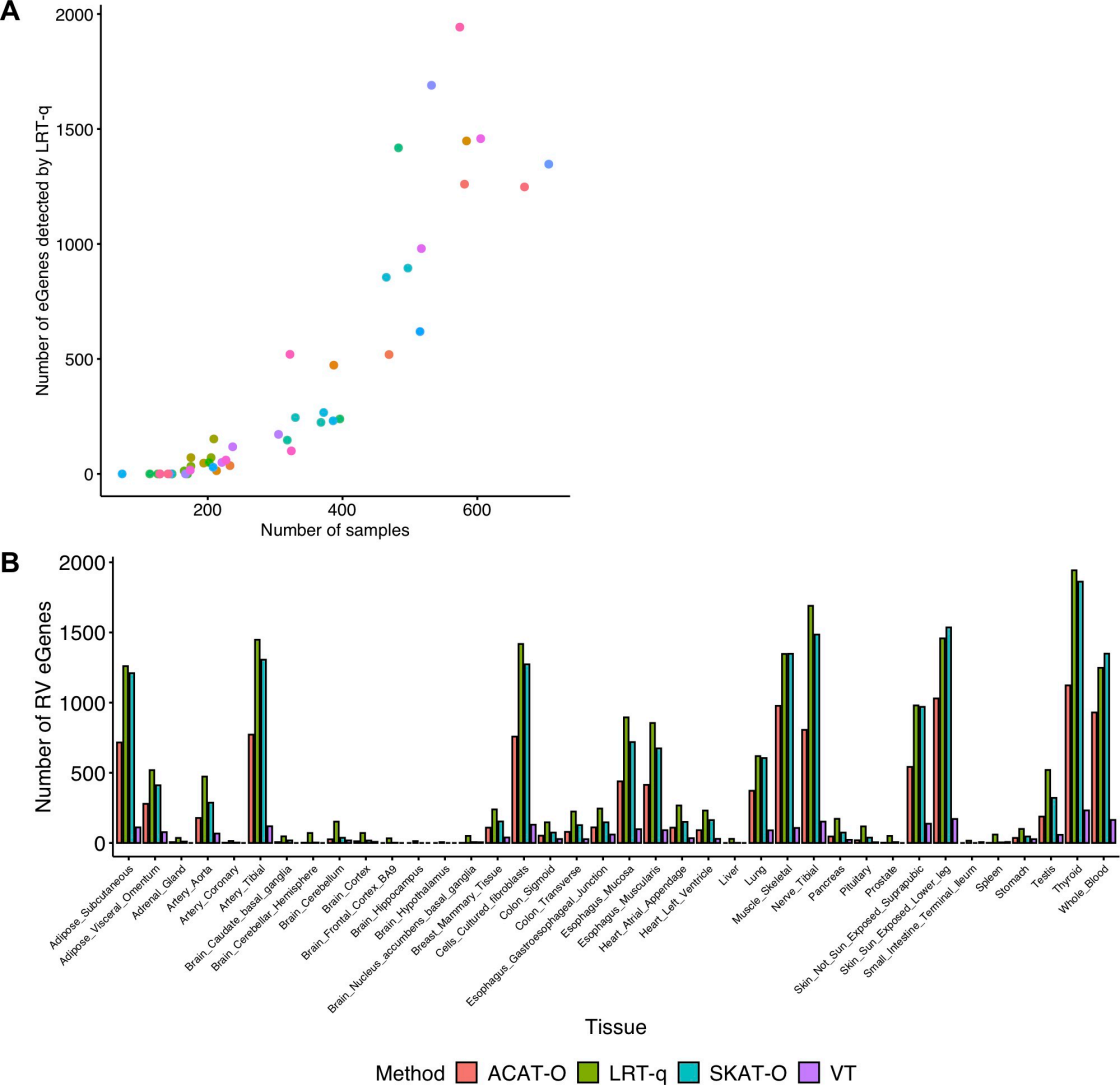
RV eGenes detected from 49 tissues in the GTEx v8 dataset. A. The relationship between the number of total RV eGenes detected by LRT-q in each tissue and the sample size of each tissue. The colors of the data points are randomly assigned. Each tissue has its own color. B. The number of total RV eGenes detected by each method. In panel B, only tissues with more than one RV eGene detected by any methods are included.

Next, we define the union set of RV eGenes identified with the eight different weighting schemes as the total set of RV eGenes detected by a method for each tissue and compare this number across different methods and tissues. First of all, as expected, the number of RV eGenes detected by LRT-q across tissues is positively correlated with sample sizes of tissues (Pearson’s *r* = 0.8966) where this phenomenon is not affected by the number of expressed genes in each tissue (Figs 2A and S6A). When comparing the number of RV eGenes detected by different methods, we find that LRT-q identifies the largest number of RV eGenes in 35 out of 41 tissues where there is at least one RV eGene detected by any method (Fig 2B) while LRT-q detects only one fewer RV eGene than SKAT-O in four tissues (Brain_Putamen_basal_ganglia, Muscle_Skeletal, Ovary, and Vagina). In three of these four tissues, including Brain_Putamen_basal_ganglia, Ovary, and Vagina, have so small sample sizes that LRT-q and VT failed to detect any RV eGenes while SKAT-O and ACAT-O identified at most one RV eGene. In general, LRT-q detects on average 308% more total RV eGenes than SKAT-O (min:1% and max:2,800%), which identifies the second most total RV eGenes in GTEx tissues. Importantly, our method identifies a few RV eGenes in tissues with small sample sizes such as brain—hypothalamus (N = 150) while other methods fail to detect any RV eGenes in these tissues. We find that our method outperforms other methods even when we lower the MAF threshold to 1% to define rare variants although we detect fewer overall RV eGenes with 1% MAF compared to those with 5% MAF, which is expected (S2 and S3 Tables). Results show that our method also discovers more novel RV eGenes than other methods in 38 out of 41 tissues, which are eGenes not reported in the GTEx v8 analysis that only considered the effects of common variants (MAF ≥ 1%). LRT-q detects only one fewer RV eGene than SKAT-O in the other three tissues (Brain_Putamen_basal_ganglia, Ovary, and Vagina) (S6B Fig). LRT-q detects on average 204% more novel RV eGenes than SKAT-O that detects the second most novel RV eGenes (min:8% and max:1,000%). These results indicate that our method detects not only more overall RV eGenes but also more novel RV eGenes that have not been discovered using common variants, which may be important in interpreting the functional effects of rare variants.

To examine overlaps among RV eGenes detected by different methods, we look at RV eGenes in four tissues, Muscle_Skeletal, Skin_Sun_Exposed_Lower_leg, Thyroid, and Whole_Blood where we have a good number of RV eGenes. We look at the overlaps of RV eGenes detected by four methods, LRT-q, SKAT-O, ACAT-O, and VT. Using the Venn diagram (S7 Fig), we find that many RV eGenes detected by SKAT-O, ACAT-O, and VT are also detected by LRT-q: on average, LRT-q detects 61.76%, 72.29%, 80.40% of RV eGenes detected by SKAT-O, ACAT-O, and VT, respectively. This result also shows that a majority of RV eGenes detected by ACAT-O and VT are shared with other methods as they detect the smallest numbers of RV eGenes. ACAT-O shares most of RV eGenes with VT and SKAT-O because it is a combination method that uses the results from SKAT-O and VT. SKAT-O also has higher proportions of shared RV eGenes with other methods compared to LRT-q, where it identifies 63.16%, 92.35%, 92.01% of RV eGenes discovered by LRT-q, VT, and ACAT-O, respectively. This result shows that LRT-q detects many RV eGenes detected by other methods and detects additional RV eGenes.

Lastly, we detect RV eGenes using independent rare variants after LD-pruning as one of the assumptions in LRT-q is the independence among variants. We find that LD-pruning increases the number of total RV eGenes and novel RV eGenes detected by LRT-q for the whole-blood tissue by 34.54% and 32.03%, respectively, using the 5% MAF threshold for rare variants. We also observe more RV eGenes for other methods (S4 and S5 Tables). This result shows that the independence assumption may limit the ability of LRT-q to detect RV eGenes, but does not increase FPR as we observe fewer RV eGenes when rare variants are not independent. For the rest of the analysis, we present the results using the 5% MAF threshold and without using LD-pruning because the number of rare variants changes considerably depending on the level of LD-pruning we perform, and it is not obvious which LD-pruning procedure yields the best results.

One important factor that may influence detection of RV eGenes is common SNP eQTLs near rare variants. It is possible that common SNP eQTLs and rare variants may be in weak LD, and LRT-q may detect the common SNP eQTL signal as a rare variant association. Note that this phenomenon does not influence our results on novel RV eGenes since they do not contain common SNP eQTLs. To identify how common SNP eQTLs may affect the detection of non-novel RV eGenes (RV eGenes that have common eQTLs), we regress out the effect of the most significant eQTL from gene expression within 20kb, 50kb, and 100kb from the transcription start sites (TSS) of each non-novel RV gene and perform rare variant association tests with LRT-q to detect RV eGenes in four tissues, including Whole_Blood, Thyroid, Muscle_Skeletal, and Skin_Sun_Exposed_Lower_leg. We select these distance ranges because we consider rare variants within 20kb from TSS while common eQTLs may be up to 1mb from TSS and we only want to regress out common eQTLs that might be in LD with rare variants. We calculate the differences in p-values of non-novel RV eGenes before and after this regression across different weights for rare variants.

The results show that p-values of most non-novel RV eGenes do not change after the regression as the median change in p-value is close to 0.0 (S8 Fig). However, we observe large changes in p-values for some non-novel RV eGenes, and hence, we decide to look at how the number of RV eGenes changes after this regression. For this, we use the fixed p-value threshold (1e-4) to identify RV eGenes instead of FDR. The reason is that we have two groups of genes: 1) genes that have significant eQTLs, and we regress out the effect of these eQTLs from gene expression, and 2) genes that do not have significant eQTLs, and hence we do not apply this regression. We find that by combing these two groups of genes, the p-value distribution changes somewhat significantly, and hence it also changes q-values significantly although the corresponding p-values have not changed much.

We find that both LRT-q and SKAT-O indeed lose some RV eGenes after regressing out the common eQTL effect, which is expected. LRT-q loses about 25.16% of RV eGenes on average where SKAT-O loses a much higher proportion of RV eGenes (36.11% on average) (S6 and S7 Tables). These results suggest that although some of the rare variant associations LRT-q detects may be due to the effect of common SNP eQTLs, they do not seem to appear very frequently. The results also suggest that for these four tissues, although LRT-q detects fewer RV eGenes (FDR < 5%) than SKAT-O before the regression except Thyroid, SKAT-O might have detected more common SNP eQTLs as RV associations as SKAT-O loses a much higher proportion of RV eGenes after the regression.

#### Patterns of tissue-shared and tissue-specific RV eGenes in GTEx

We investigate tissue-sharing patterns of RV eGenes in GTEx to determine whether related tissues share more RV eGenes and to compare these patterns to those from CV eGenes, which are eGenes detected from common variants in the previous GTEx analysis. To find a tissue-sharing pattern of RV eGenes between a pair of tissues, we count the number of RV eGenes shared between the two tissues and divide it by the number of RV eGenes in the tissue with fewer RV eGenes. It is important to note that this approach is different from the previous GTEx analysis that used the correlation of effect sizes of common eQTLs between a pair of tissues. As calculating the combined effect size of rare variants is not obvious, we instead calculate the fraction of RV eGenes shared between a pair of tissues and apply the same approach to CV eGenes for comparison. Lastly, as some tissues have very few RV eGenes, we use FDR of 10% to increase the number of RV eGenes in each tissue.

**Fig 3:**
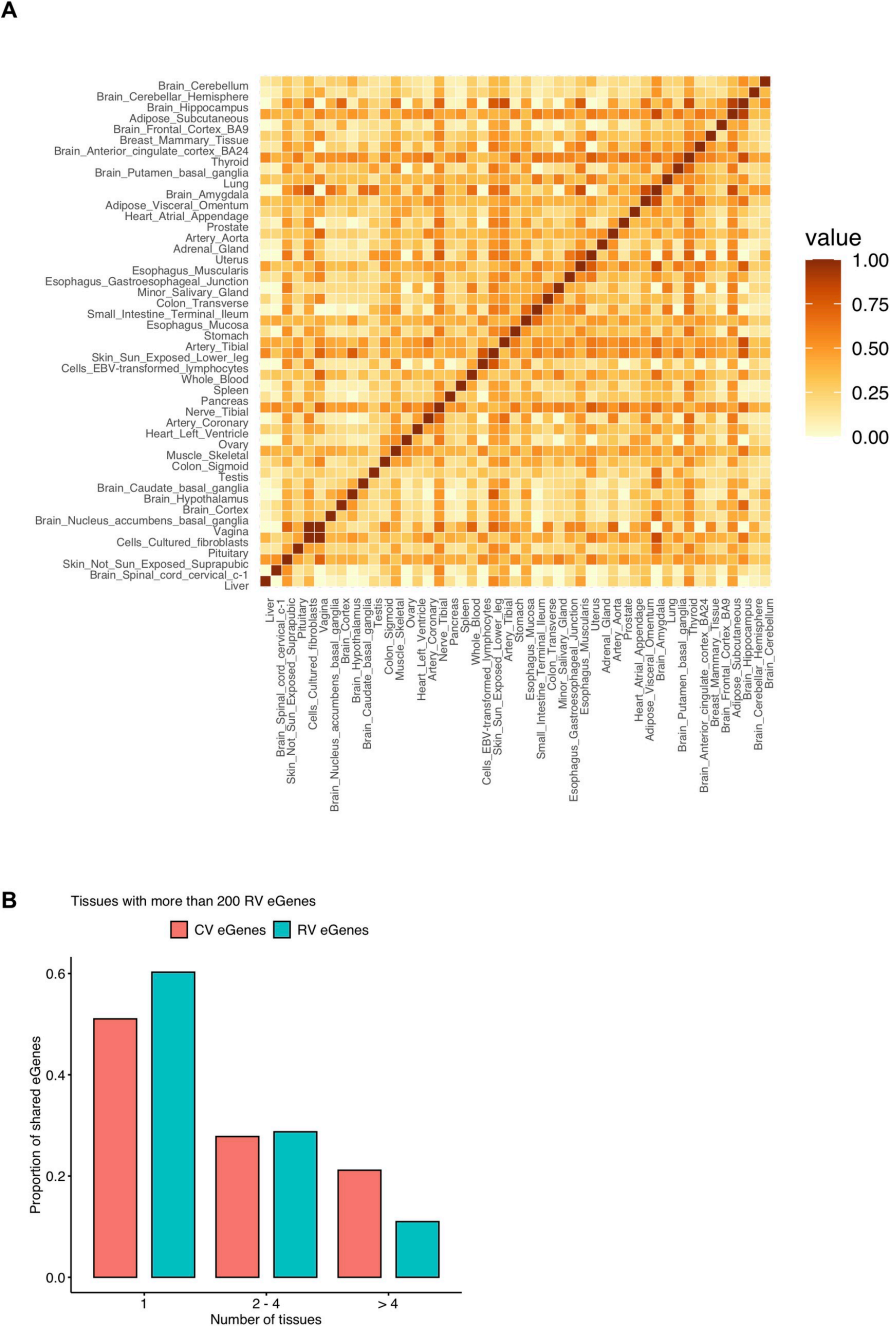
Tissue-sharing patterns of RV eGenes in the GTEx v8 dataset. A. Pairwise tissue-sharing matrix of RV eGenes. It shows the fraction of shared RV eGenes in each pair of tissues. Here we use FDR < 10% to increase the number of RV eGenes. Tissues are sorted by clustering. Only tissues with more than one RV eGenes are included. B. The proportion of RV eGenes and CV eGenes shared among different numbers of tissues. Only tissues with more than 200 RV eGenes are selected. It shows the proportion of eGenes that are only detected in one tissue, in 2–4 tissues, and in more than 4 tissues.

We observe that tissues with related functions share a high fraction of their RV eGenes and are clustered together, such as most brain tissues (11 out of 12 brain tissues) and tissues in the digestion system including stomach, esophagus, colon, and small intestine tissues (Fig 3A). Also, there are a few related tissues that share a high fraction of RV eGenes (S9 Fig). For example, three artery tissues share on average 58.52% of RV eGenes among them, esophagus—muscularis and esophagus—gastroesophageal junction share 53.32% of RV eGenes, and two skin tissues share 48.62% of RV eGenes. The overall tissue sharing patterns of RV eGenes are similar, although attenuated, to patterns of tissue sharing of CV eGenes; we observe stronger tissue sharing patterns of functionally related tissues for CV eGenes (S10A and S11 Figs). Interestingly, we observe two separate clusters of brain tissues in the tissue-sharing matrix (Fig 3A), as the patterns of RV eGenes sharing among brain tissues are not strong where the average fraction of RV eGene sharing is 27.75%, compared to CV eGenes where the average fraction of CV eGene sharing is 58.85%. This may be due to the small numbers of RV eGenes detected in those tissues, where there are 34.08 RV eGenes on average for each brain tissue compared to 6870.15 CV eGenes.

Next, we identify a pattern of tissue-sharing across more than two tissues, and for this analysis, we use 20 tissues that have at least 200 RV eGenes as tissues with only a few RV eGenes would not share many eGenes with other tissues. Among 7,857 unique RV eGenes in those 20 tissues, we find that 60.26% of them are RV eGenes in only one tissue (“tissue-specific”), 28.74% of them are RV eGenes in 2–4 tissues, and only 11.00% of them are eGenes shared in more than 4 tissues (Fig 3B). To compare this result with the tissue-sharing pattern of CV eGenes, we select the top *N*_*t*_ of CV eGenes sorted by FDR q-values from tissue *t* where *N*_*t*_ is the number of RV eGenes in tissue *t*, so that we compare the same number of CV and RV eGenes from each tissue. This is necessary to make tissue-sharing patterns of CV and RV eGenes comparable as there are many more CV eGenes than RV eGenes in general and many CV eGenes are shared across many tissues without this selection of top CV eGenes. We observe a different pattern of tissue-sharing of top CV eGenes where CV eGenes are less tissue-specific than RV eGenes; 51.04% of CV eGenes are tissue-specific compared to 60.26% of RV eGenes, and 21.15% of CV eGenes are shared in more than 4 tissues, which is about twice higher than the fraction of RV eGenes shared in that number of tissues (Fig 3B). We repeat this experiment selecting 25 tissues with at least 100 RV eGenes and observe similar results (S10B Fig). These results demonstrate that the tissue-sharing patterns of RV eGenes reflect the functional relationship among tissues, and they tend to be more tissue-specific when compared to CV eGenes.

#### Enrichment of expression outliers, proximal rare variants, and disease-associated genes among RV eGenes in GTEx

Previous studies have shown that large-effect regulatory rare variants may cause abnormal gene expression, causing individuals carrying those variants to have significantly higher or lower expression for certain genes[14] In this analysis, we investigate whether RV eGenes we detect from LRT-q are enriched with expression outliers who have abnormal gene expression compared to other non-RV eGenes. First, similar to Li et al.[14], we correct gene expression measurements for age, sex, genotyping principal components, and PEER factors, and then generate standardized Z-scores. We define expression outliers as individuals with standardized gene expression |Z-score| > 2 and count the number of outliers in each gene. Because genes may be expressed differently depending on tissues, outliers are defined specific to genes and tissues. In each tissue, we count the number of outliers for each RV eGene and non-RV eGene separately, and then we aggregate these counts across all tissues. We observe that 8,090 unique RV eGenes (FDR < 5%) across all 49 tissues have 19.52 expression outliers on average, which is significantly greater than 13.28 outliers on average in 30,436 non-RV eGenes (t-test p < 2.2e-16). We also look at whether those expression outliers carry rare variants within 20K bp of a TSS of each eGene, and we find that across all tissues, on average, 72.17% of expression outliers carry one or more rare variants (S12 Fig).

**Fig 4:**
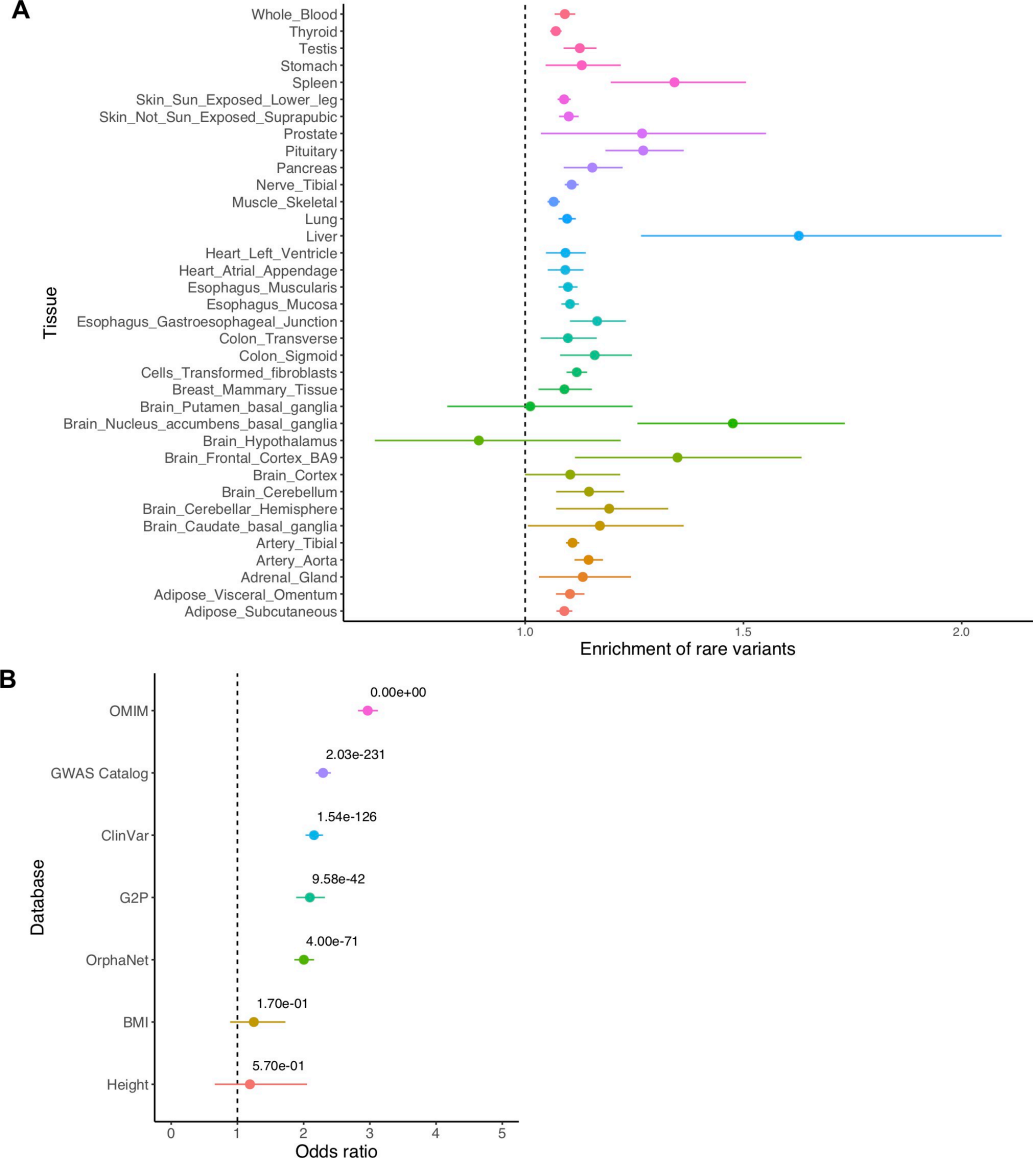
Outlier analysis of RV eGenes detected by LRT-q in GTEx v8. A. Enrichment of proximal rare variants in outliers compared to non-outliers for RV eGenes in each tissue. Tissues without RV eGenes are excluded. B. Enrichment of RV eGenes in disease-associated genes and genes related to common traits (BMI and Height) from public databases. The numbers represent p-values. In both panels, we show the mean values as dots and 95% confidence intervals as error bars.

Li et al. discovered that expression outliers were enriched for rare variants near the TSS compared to non-outliers, and we investigate whether this enrichment is stronger for RV eGenes compared to non-RV eGenes. This enrichment is defined as the ratio between the proportion of outliers with proximal rare variants (those within 20kb of TSS) and the proportion of non-outliers with the rare variants for each gene, which can be thought of as relative risk of carrying the rare variants in outliers vs. non-outliers. Using FDR of 5% to detect RV eGenes in each tissue, our results show that outliers are significantly enriched for proximal rare variants compared to non-outliers in all tissues except three brain tissues (Brain_Putamen_basal_ganglia, Brain_Hypothalamus, and Brain_Cortex) with limited sample sizes (Fig 4A). For non-RV eGenes, we do not observe this enrichment in all tissues. We observe consistent results when varying Z-score thresholds to define expression outliers; outliers are significantly enriched for adjacent rare variants compared to non-outliers regardless of Z-score thresholds and the enrichment increases as the Z-score thresholds increase (S13 Fig). These results suggest that rare variants with *cis-*regulatory effects may be key factors to explain the large changes in gene expression levels and those rare variants are likely to have significant contributions to RV eGenes.

Lastly, we hypothesize that RV eGenes are more likely to be associated with diseases or traits. For this analysis, we calculate enrichment of RV eGenes among five online disease gene databases (see Materials and Methods) including 6,298 genes from NCBI ClinVar database[34], 2,569 genes from Genotype-to-Phenotype (G2P) database[35], 20,998 reported GWAS genes from NHGRI-EBI catalog[1], 26,352 genes from Online Mendelian Inheritance in Man (OMIM) database[36], and 7,298 genes from OrphaNet database[37]. We choose these databases because they facilitate the development, curation, validation of large-scale datasets for associations between human genetic variants and complex and rare diseases. We also consider genes for non-disease traits as positive controls, which are 212 genes related to body mass index (BMI) and 78 genes related to height that are provided by the GeneRIF database and downloaded from the Harmonizome database[38]. Note that GeneRIF is a public database for the functional annotations of genes based on previous literature. We construct a 2x2 contingency table where an outcome is whether a gene is a disease gene for each database and an exposure is whether a gene is an RV eGene or a non-RV eGene. We use 8,090 RV eGenes we detect from all 49 tissues with FDR of 5%, and p-value is computed with the Fisher’s exact test where odds ratio (OR) greater than 1 indicates enrichment of RV eGenes in a disease database while OR less than 1 indicates depletion. Results show that RV eGenes are significantly enriched in five disease databases with OR ranging from 2.00 to 2.97 (p = 0~9.58e-42, Fig 4B), and the largest OR is observed in the OMIM database that contains genes involved in Mendelian disorders. We also observe an odds ratio of around 1.0 for genes related to BMI and height, as expected, because they are two common traits and are not related to any certain diseases. These results suggest that RV eGenes are much more likely to be involved with diseases compared to non-RV eGenes while they are not enriched in non-disease traits.

#### Analysis of disease-associated RV eGenes

To discover the clinical importance of RV eGenes we identify, we perform a literature search on RV eGenes using the ClinVar database. Specifically, we attempt to find whether an RV eGene in a specific tissue is associated with a certain disease related to that tissue. First, we find that patients with platelet count disorders carry rare variants in *TUBB1*[39] where *TUBB1* is detected by our method as one of the RV eGenes in the heart left ventricle and skin tissue. The landmark symptom of platelet count disorders is petechiae on the skin[40]. There is one rare variant (rs41303899) in *TUBB1* that was reported as likely pathogenic for this disorder where the gnomAD frequency for this variant is 1.5E-3 in the European population. Interestingly, one individual in GTEx carries this rare variant although the disease status of this individual is not available. We find that the adjusted *TUBB1* expression Z-score of this individual carrying this rare variant is 1.15 in skin tissue, which is relatively high.

Another example of an association between RV eGenes found in a particular tissue and a tissue-specific disease caused by rare variants in those genes is telomere sheltering gene *POT1*, which is one of the RV eGenes found in fibroblasts. Fibroblasts were found to contribute to the growth and drug resistance of melanoma, a potentially lethal form of skin cancer[41]. Previous whole-exome sequencing studies found that rare variants in *POT1* could increase the risk for familial cutaneous malignant melanoma, as one of the rare variants, rs587777477, was discovered to perturb telomere maintenance[42,43]. We also find that *IFIH1* is identified as an RV eGene in skin tissue, and the rare missense variant, rs587777446, in this gene has been shown to be pathogenic for autosomal dominant inflammatory disorder, Aicardi-Goutieres syndrome 7 with the phenotype of skin swelling[44,45]. These examples indicate that some RV eGenes are associated with diseases caused by rare variants in relevant tissues, which demonstrates the clinical importance of RV eGenes.

## Discussion

We have proposed LRT-q as a powerful rare variant association test for identifying the effects of rare variants on gene expression. Our simulation studies showed that the proposed method had a well-controlled false positive rate and higher statistical power compared with other methods. Through the analysis of gene expression data of 49 tissues from the GTEx dataset, we demonstrated that LRT-q detected more genes whose expression was regulated by nearby rare variants, which we call RV eGenes, compared to other approaches including SKAT-O. More importantly, our method discovered the largest number of novel RV eGenes that were not regulated by common variants reported in GTEx, which might be particular interest to studies analyzing the functional effects of rare noncoding variants. These results show that LRT-q is an effective statistical method for rare variant association analyses for quantitative traits including gene expression.

RV eGenes discovered from 49 tissues in GTEx provided several important biological insights about gene regulation of rare variants. First, we found that as expected, a pair of functionally related tissues shared a high proportion of RV eGenes because their gene expression values were correlated. However, the levels of tissue-sharing patterns of RV eGenes were not as high as those of CV eGenes where one main reason is the limited number of RV eGenes compared to the number of CV eGenes. We detected far fewer RV eGenes than CV eGenes with the same sample size, which is also expected as we have lower power to detect the effects of rare variants than common variants even with the rare variant association methods that combine effects of multiple rare variants[5,46,47]. This suggests that we need larger sample sizes to detect more RV eGenes.

Next, when we checked the tissue sharing patterns of RV and CV eGenes across 20 tissues using the same number of RV and CV eGenes for each tissue, we found that a higher proportion of RV eGenes were detected only in one tissue than CV eGenes where CV eGenes had a much higher proportion of genes shared across more than four tissues. This suggests that the effects of rare variants on gene expression may be more tissue-specific than common variants, which is important in interpreting results of rare variant associations for complex diseases and traits. However, we anticipate that a higher fraction of RV eGenes will be shared across many tissues as more RV eGenes are discovered with a larger sample size as we have observed this phenomenon with CV eGenes[48,49].

Lastly, we explored the characteristics of RV eGenes with a series of enrichment analyses. We found that all RV eGenes had several outliers whose expression levels deviate significantly from the rest of the individuals. These outliers had enrichment of rare variants near the TSS of RV eGenes compared to non-outliers while there was no such enrichment for non-RV eGenes, suggesting that rare variants carried by outliers may play important roles in causing the abnormal expression levels of the outliers for RV eGenes. Additionally, we discovered that RV eGenes were significantly enriched for disease-associated genes across all human disease databases, indicating that genes whose expression is influenced by nearby rare variants have a higher chance of being associated with diseases. Moreover, previous findings provided evidence supporting that rare mutations in our RV eGenes could increase the risk for certain diseases in the same tissues where they were discovered. This further suggests that rare variants with regulatory effects may help identify genes associated with diseases.

There are four main features of LRT-q that make it a highly powerful test as demonstrated in simulation and the GTEx data. First, it prioritizes potential causal rare variants with genotype data and functional information such as CADD scores. With our formulation of the likelihood ratio test, LRT-q attempts to find the most likely scenario of causal statuses of rare variants, which increases the power of detecting potential causal variants. Second, it applies nonlinear aggregation of rare variants, which results in a nonlinear decision boundary in detecting their effects. Using simulations, we show that the nonlinear decision boundary enables LRT-q to emphasize the effects of causal variants in its test statistic, leading to a higher power. Third, as an extension of the original LRT method, LRT-q also computes the likelihoods for all possible scenarios of causal statuses using an efficient decomposition technique, which reduces the computational complexity and enables LRT-q to be applied to large-scale datasets. Fourth, LRT-q considers both directions of rare variant effects as LRT-q statistics are based on the normal distribution that considers the absolute values of effect sizes and not their directions. This is important because some regulatory variants may increase gene expression (positive effect) while other variants may decrease it (negative effect). Results from the real GTEx data appear to suggest that rare variants are likely to have different directions of effect because VT, a method that assumes the same direction of effects of rare variants, detected much fewer RV eGenes than LRT-q; if variants had consistent directions of effect, VT would have detected many more RV eGenes.

The application of LRT-q can be extended to other quantitative traits, such as height and BMI. As the likelihood ratio test is the most powerful test for a particular hypothesis test according to the Neyman-Pearson lemma[50], one is likely to achieve higher power with LRT-q on other quantitative traits than other previous methods. Also, LRT-q can be generalized to a gene-based test or a region-based test as well as analysis of gene sets, pathways, or networks. It is, however, important to find appropriate weights for rare variants because results may change considerably depending on those weights as our results showed that different functional annotations of rare variants affected power to detect RV eGenes. To address this issue, we used a straightforward approach that employs a variety of functional annotations and combines results. Identifying an ideal set of weights for rare variants for gene expression and an optimal approach to combine results remains an important research topic. One of the limitations of LRT-q is that it may be computationally demanding as it needs to perform a permutation test to estimate p-value for each gene. One approach to improve the efficiency of LRT-q is performing an adaptive permutation test that stops the permutation test when observing p-values from a small number of permutations are high (e.g., > 0.05). Assuming that most genes are not RV eGenes, we would only need to perform 1,000 or fewer permutations for the majority of genes. For those genes with small p-values (potentially RV eGenes), we would perform up to 100,000 permutations to obtain more accurate p-values. We find that the adaptive permutation test yields a similar number of RV eGenes compared to the permutation test that uses 100,000 permutations (S14 Fig).

For efficient calculation of LRT-q statistic and the corresponding p-value, we assume the independence between rare variants as previous studies[28,51] have found that there would be very low LD among rare variants. In this study, we found by performing LD-pruning that violation of the assumption of independence between rare variants may reduce the power of LRT-q, but it does not increase FPR. In our analysis of the GTEx data, we did not perform LD-pruning as the optimal LD-pruning approach is not currently known for rare variants. Researchers may want to apply LRT-q to their eQTL data after applying LD-pruning to identify more RV eGenes.

## Materials and methods

### LRT-q model

Suppose that we have genotype and gene expression data of a population with size *N*, and perform an association test for a gene with *k* rare variants. For rare variant *i* (1≤*i*≤*k*), there are *m*_*i*_ individuals not carrying a rare variant (e.g. not carrying a minor allele of a rare variant), whose expression levels are $X_{i}=\{x_{i}^{1},x_{i}^{2},\cdots ,x_{i}^{m_{i}}\}$, and *n*_*i*_ subjects carry a rare variant *i* (e.g. carrying a minor allele), whose expression levels are $Y_{i}=\{y_{i}^{1},y_{i}^{2},\cdots ,y_{i}^{n_{i}}\}$. Note that *N* = *m*_*i*_+*n*_*i*_. There are two assumptions in our model: 1) independence among rare variants (e.g. no linkage disequilibrium (LD) among rare variants) and 2) the normality of gene expression values. Previous studies have suggested that there would be very low linkage disequilibrium (LD) among rare variants because of their low frequencies[28,51]. As for the normality assumption, gene expression values are often quantile normalized in eQTL studies[48,49,52,53], which means that *X*_*i*_ and *Y*_*i*_ can be viewed as random samples from a standard normal distribution. With these two assumptions, *X*_*i*_ and *Y*_*i*_ are independently and normally distributed ($X_{i}∼N(\mu_{X_{i}},\sigma_{X_{i}}^{2}),Y_{i}∼N(\mu_{Y_{i}},\sigma_{Y_{i}}^{2})$, where $\mu_{X_{i}},\mu_{Y_{i}}$ stand for the means of *X*_*i*_, *Y*_*i*_ and $\sigma_{X_{i}},\sigma_{Y_{i}}$ represent the standard deviation of *X*_*i*_, *Y*_*i*_, respectively). We are interested in testing the effects of rare variants on gene expression, that is, the difference between *X*_*i*_ and *Y*_*i*_. Thus, we test the following hypotheses

$$H_{0}:\forall 1\leq i\leq k,\mu_{X_{i}}=\mu_{Y_{i}}\;versus\;H_{1}:\exists 1\leq i\leq k,\mu_{X_{i}}\neq \mu_{Y_{i}}$$

The null hypothesis (*H*_0_) asserts that no rare variants have regulatory effects, while the alternative hypothesis (*H*_1_) states that there is at least one causal rare variant affecting gene expression. Here, $\sigma_{X_{i}}$ and $\sigma_{Y_{i}}$ are both unknown but assumed to be equal to the pooled variance *σ*_*i*_.

To boost the statistical power, we want to infer which rare variants are causal. Here, let *v*_*i*_ be an indicator variable for the causal status of variant *i* (1≤*i*≤*k*); *v*_*i*_ = 1 if variant *i* is causal and 0 otherwise. Let *V* = {*v*_1_, *v*_2_,….,*v*_*k*_} be the causal statuses of *k* rare variants. Then there are 2^*k*^ possible values for *V*, because each of the *k* rare variants can have causal effects on gene expression or not. Among them, let $V_{q}=\{v_{1}^{q},v_{2}^{q},\cdots ,v_{k}^{q}\}$ be the *q*^*th*^ vector, representing a specific scenario of causal status. Using the functional information on rare variants, such as CADD scores, we can obtain the probability of variant *i* being causal *c*_*i*_ = *P*(*v*_*i*_ = 1). Using the assumption that rare variants are independent, the probability of each scenario *V*_*q*_ is given by

$$P(V_{q})=\prod_{i=1}^{k}c_{i}^{v_{i}^{q}}(1-c_{i}{)}^{1-v_{i}^{q}}$$ (1)

We calculate the likelihood of the observational data and the inferred causal statuses *V*_*q*_ as follows

$$L(X,Y,V_{q})=L(X,Y|V_{q})P(V_{q})$$ (2)

where *X* = *X*_1_, *X*_2_,⋯,*X*_*k*_, *Y* = *Y*_1_, *Y*_2_,⋯,*Y*_*k*_ are gene expression levels of individuals without rare variants and with rare variants, respectively. This equation considers both observational data (gene expression and genotype data) and causal statuses of rare variants, and therefore can prioritize causal variants by functional information. We then calculate our statistic as the ratio between the likelihood under the null hypothesis and the likelihood under the alternative hypothesis and use a permutation test to compute the p-value. More detailed information on the derivation of likelihood ratio test, its decomposition for efficient calculation of the test statistic, and parameter estimation is discussed in S1 Text.

### Simulation studies

To compare the performance of LRT-q with the widely used existing rare variant association tests, we measure their type I error rates and statistical power in simulation studies. In this study, data are simulated with a similar framework described in Wu et al.’s work[23].

#### Simulation of genotype data

The calibration coalescent model[54] (COSI) is used to generate 50,000 haplotypes, assuming that they have the LD structure of individuals of European ancestry. Any pairs of haplotypes could be combined into diplotypes. In each replicate, a 5 kb region is randomly selected to simulate the diplotypes for 1,000 individuals, which contains 33.1 rare variants (MAF < 0.05) on average. We also perform the power simulation with a 3 kb region including 19.8 rare variants on average.

#### Type I error rate simulation

Under the null hypothesis of no association between rare variants and gene expression, we simulate the normalized expression levels for individual *j* from the model described as follows.

$$E_{j}=A_{j}+\epsilon_{j}$$

where *A*_*j*_~*N*(0,1) represents the covariates and *ϵ*_*j*_~*N*(0,1) stands for random errors. Each *E*_*j*_ is assumed to be independent. We simulate 100,000 replicates to test the type I error rate at the significance level *α* = 0.05,0.01,0.001,0.0001. When applying rare variant association methods to the datasets, we use uniform weights for all rare variants (e.g. assuming all rare variants are likely causal). VT and LRT-q are run with 10,000 permutations to measure p-values.

#### Power simulation

Under the alternative hypothesis where there is at least one causal rare variant influencing gene expression, we use the following model to simulate the gene expression value for individual *j*.

$$E_{j}=A_{j}+\beta^{T}G_{j}+\epsilon_{j}$$

where *A*_*j*_~*N*(0,1) represents the covariates of individual *j* and *ϵ*_*j*_~*N*(0,1) stands for random errors. Here, we randomly sample *s* variants out of the total *k* rare variants as causal variants. *G*_*j*_ = (*g*_*j*1_,…,*g*_*js*_) is defined as genotypes of *s* causal rare variants of individual *j* where *g*_*ji*_ = 0,1,2 depending on the number of rare alleles for variant *i*. Their effect sizes are set to be *β* = *a*|*log*_10_*MAF*|, where *MAF* represents the minor allele frequencies of causal rare variants and *a* is a constant. In this study, *a* is set to be a fixed constant 0.3, 0.6, 0.9, 1.2, or 1.5 and we also assume 3%, 5%, 7%, or 10% of rare variants to be causal to simulate different numbers of causal variants with different effect sizes. Thus, in this simulation study, the maximum effect size of a causal rare variant would be 0.99, 1.98, 2.97, 3.96, or 4.95 assuming 1,000 individuals and a MAF cutoff of 5%. Causal rare variants have 50% probability of having negative effect sizes (e.g. decrease gene expression), and 50% probability of having positive effect sizes (e.g. increase gene expression). The statistical power is estimated as the proportion of p-values smaller than *α* = 0.05 in 10,000 simulated datasets. Similar to the type I error simulations, all rare variants are weighted equally. Both LRT-q and VT are run with 1,000 permutations to calculate p-values. We also generate simulations to visualize decision boundaries of LRT-q, SKAT-O, and CMC approaches, and a detailed description of this simulation is discussed in S1 Text.

To examine the robustness of the LRT-q model, we generate simulations using different settings. First, we sample *a* from *N*(1,1) to add random noises to effect sizes of rare variants. Second, we perform the power simulation with fewer rare variants in a gene. Third, we simulate *X*_*i*_ (gene expression levels of individuals not carrying the rare variant *i*) and *Y*_*i*_ (gene expression levels of individuals carrying the rare variant *i*) to have different variances, which violates the assumption of LRT-q model in parameter estimation. Here, we let *X*_*j*_ = *A*_*j*_+*ϵ*_*j*_, where *A*_*j*_~*N*(0,1) represents the covariates and *ϵ*_*j*_~*N*(0,1) stands for random errors. Let *Y*_*j*_ = *B*_*j*_+*β*^*T*^*G*_*j*_+*ϵ*_*j*_, where *B*_*j*_~*N*(0,2) represents the covariates, *ϵ*_*j*_~*N*(0,1), and *β*^*T*^*G*_*j*_ stands for the effects of causal rare variants. Hence, X and Y have different variances.

### Analysis of multi-tissue GTEx v8 WGS and RNA-seq data

We download the GTEx dbGaP release v8 RNA-seq data from the GTEx portal and the whole-genome sequencing (WGS) data from dbGaP accession number phs000424.v8.p2. Genotype data and transcriptome data from all 49 GTEx tissues are used in this study. There are 838 subjects with both WGS and RNA-seq data.

#### Quality control

We identify 681 individuals of European ancestry using EIGENSTRAT[55]. We consider only Europeans because they are the largest homogenous population in GTEx. We then extract only European samples from each tissue, creating 49 separate genotype datasets for the 49 tissues. We restrict our analysis to autosomal variants. For these 49 genotype datasets, we extract rare single nucleotide variants (SNVs), which are defined as variants with minor allele frequency (MAF) < 5%; we also test a case when rare variants have MAF < 1%. Alleles with genotyping quality (GQ) less than 20 are marked missing. We remove variant sites that have a missing rate larger than 5% or failed variant quality score calibration (VQSR)[56]. Then missing genotypes are imputed as two reference alleles because of the low frequency of rare variants.

#### RV eGene discovery in the GTEx dataset

SNVs are functionally annotated with CADD[26] and LINSIGHT[32]. Next, we group variants in a gene and those located within 20kb upstream or downstream of transcription start site (TSS) of a gene. The summary statistics of sample size, the number of genes and rare variants for each tissue after preprocessing is in S8 Table. Covariates of each sample provided by GTEx, which are top 5 genotyping principal components, PEER factors[57] (15 factors for tissues with fewer than 150 samples, 30 factors for those with 150–250 samples, 45 factors for those with 250–350 samples, and 60 factors for those with more than 350 samples), sequencing platform, and sex are used to regress out unwanted confounding effects in gene expression levels for each tissue using a linear model. Then the transformed gene expression levels are normalized with rank-based inverse normal transformation using “RankNorm” in the “RNOmni” R package. When applying rare variant association methods to the GTEx data, different weighting strategies of rare variants are used, including LINSIGHT scores, CADD scores, MAF, distance to TSS, and uniform weights (all rare variants have the same weight). Note that weighting by MAF or TSS distance is to use weights inversely proportional to the values of MAF or TSS distance, so variants with lower frequency or closer to TSS are assigned higher weights while for other weightings, higher scores (e.g. CADD or LINSIGHT scores) mean higher weights for variants. We also combine multiple weights by multiplying two or three weights together for each variant; we create three combined weights, 1) MAF × TSS distance, 2) MAF × CADD scores, and 3) MAF × CADD scores × TSS distance. All weights mentioned above are employed to discover RV eGenes in 49 GTEx tissues. FDR < 5% is applied for multiple testing correction.

#### Patterns of tissue sharing in RV and CV eGenes

We first assess tissue-sharing patterns of RV eGenes in a pair of GTEx tissues. We use FDR < 10% to identify RV eGenes in each tissue to increase the RV eGenes. Next, for each pair of tissues, we calculate the fraction of shared RV eGenes as

$$\frac{\#of\;shared\;RV\;eGenes\;between\;tissues\;1\;and\;2}{min(\#of\;RV\;eGenes\;in\;tissue\;1,\#of\;RV\;eGenes\;in\;tissue\;2)}$$

Similarly, to calculate pairwise tissue-sharing patterns of CV eGenes, we select CV eGenes with FDR < 5% based on the summary statistics in the GTEx v8 dataset and calculate the fraction of shared CV eGenes using the same equation. To assess tissue-sharing patterns of RV eGenes in more than two tissues, we choose 20 tissues with at least 200 RV eGenes (FDR < 5%) and calculate the proportion of RV eGenes shared across different numbers of tissues (i.e. # of RV eGenes present in only one tissue, in 2–4 tissues, or in more than 4 tissues). To find tissue-sharing patterns of CV eGenes in more than two tissues among the same 20 tissues, we choose top *N*_*t*_ CV eGenes from tissue *t* where *N*_*t*_ is the number of RV eGenes in tissue *t*. We then calculate the proportion of CV eGenes shared across different numbers of tissues. We also repeat this analysis with another group of 25 tissues that have more than 100 RV eGenes (FDR < 5%).

#### Single-tissue gene expression outlier discovery

For each individual, we log-transform gene expression value as *log*_2_(*TPM*+1) for each gene and each tissue, where TPM is the number of transcripts per million RNA molecules. We then standardize gene expression value for each gene in each tissue into Z-score to avoid the shrinkage of outlier gene expression caused by rank-based quantile normalization, using the following equation:

$$Z_{gj}^{t}=\frac{x_{gj}^{t}-\mu_{g}^{t}}{\sigma_{g}^{t}}$$

where $x_{gj}^{t}$ and $Z_{gj}^{t}$ represent unstandardized log-transformed gene expression value and the standardized Z-score of individual *j* for gene *g* in tissue *t*, respectively. $\mu_{g}^{t}$ and $\sigma_{g}^{t}$ are the mean and standard deviation of the unstandardized values across all individuals ($x_{gj}^{t}$), for gene *g* in tissue *t*, respectively. Next, for each gene in each tissue, we regress out the covariates, including top 5 genotyping principal components, PEER factors, sequencing platform, and sex from the transformed and standardized gene expression values using a linear model. The resulting regression residuals are standardized again using the equation above and the resulting Z-scores are used to determine outliers.

Single-tissue gene expression outliers in a gene are defined as the individuals with extreme gene expression levels who have |Z-score| > 2, while the remaining individuals are defined as non-outlier for this gene. Other Z-score thresholds are also tested, including 1, 3, 4, 5, 6, 7, 8, 9, and 10. Under this definition, an outlier is specific to a gene in a certain tissue. Therefore, each gene may have different sets of outliers across tissues, and an individual may be an outlier for multiple genes in one or more tissues. We analyze all outliers in non-RV eGenes and RV eGenes identified by LRT-q in 41 out of all 49 tissues with at least one RV eGene (FDR < 5%).

#### Enrichment analysis of RV eGenes

To calculate enrichment of proximal rare variants near RV eGenes in gene expression outliers compared to non-outliers, we consider rare variants (MAF ≤ 5%) within 20 kb of the TSS of a gene. Similar to the analysis conducted by Li et al.[14], enrichment is defined as the ratio of the proportion of outliers carrying rare variants to the corresponding proportion of non-outliers. It is equivalent to the relative risk of having proximal rare SNVs as an outlier. The 95% Wald confidence intervals are calculated with the asymptotic distribution of the log relative risk. We also assess this enrichment by varying Z-score thresholds to define expression outliers (from 1 to 10). Enrichment is similarly calculated for non-RV eGenes.

We also examine the enrichment of RV eGenes for disease- or trait-associated genes in five public databases, including 6,298 genes from NCBI ClinVar database[34], 2,569 genes from Genotype-to-Phenotype (G2P) database[35], 20,998 reported GWAS genes from NHGRI-EBI catalog[1], 26,352 genes from Online Mendelian Inheritance in Man (OMIM) database[36], and 7,298 genes from OrphaNet database[37], We also consider genes related to two non-disease traits, 212 genes related to BMI and 78 genes related to height that are provided by the GeneRIF database and downloaded from the Harmonizome database[38]. We construct a 2x2 contingency table where an outcome is whether a gene is a disease gene for each database and an exposure is whether a gene is an RV eGene or a non-RV eGene. Odds ratios and 95% confidence intervals are computed by applying Fisher’s exact test to compare non-RV eGenes and RV eGenes to each of the five lists of disease- or trait-associated genes.

#### Analysis of disease-associated RV eGenes

To find evidence supporting the clinical importance of the identified RV eGenes, we do literature research in the ClinVar database. We search the database for the information about known relationships between rare variants in RV eGenes and observed health status. The information includes diseases, tissues, clinical significance, variants and their frequencies, and supporting literature.

## Supporting information

S1 Text Supplemental methods. It describes the mathematical model of the likelihood ratio test used in LRT-q, the derivations of the equations for parameter estimation in the model, and the decision boundary simulation framework.
(DOCX)

S1 Fig Power comparison among different methods on simulated data with different settings, under the significance level α = 0.05. We sample *a* from a normal distribution *N*(1,1) and simulate genotypes with (A) 19.8 rare variants on average and (B) 33.1 rare variants on average. We also simulate *X* and *Y* to have explicitly different variances with *a* sampled from a normal distribution *N*(1,1), and then perform association tests on the simulated genotypes with (C) 19.8 rare variants on average and (D) 33.1 rare variants on average.
(TIF)

S2 Fig Power simulation with 19.8 rare variants on average, assuming different variances of *X* and *Y*. A. for different effect sizes and fixed causal ratio (10%), and for fixed effect sizes (B. ≤ 0.99, C. ≤ 2.97, D. ≤ 4.95) and various causal ratios. Significance level α = 0.05.
(TIF)

S3 Fig Power simulation with 33.1 rare variants on average, assuming different variance of *X* and *Y*. A. for different effect sizes and fixed causal ratio (10%), and for fixed effect sizes (B. ≤ 0.99, C. ≤ 2.97, D. ≤ 4.95) and various causal ratios. Significance level α = 0.05.
(TIF)

S4 Fig Decision boundaries of four methods, under the significance level α = 0.05. It shows the decision boundary of A. LRT-q, B. SKAT-O, C. VT, D. CMC at *c*_*i*_ = 0.5. *c*_*i*_ represents the probability of a rare variant being causal. Each data point is labeled as significant if a significant association is identified, or not significant otherwise. "CE = 0": there are no effects of causal variants in the dataset because there are no causal variants. "CE > 0": there are effects of causal variants in the dataset because there are at least one causal variants. We randomly sample 2,500 data points to show to avoid overplotting.
(TIF)

S5 Fig The number of rare variants within 1 Mb and 20 Kb from a transcription start site (TSS) of each gene. It shows the distribution of the number of rare variants in a window of 1 Mb (median: 15,482) and 20 Kb (median: 311) around TSS across all genes in the GTEx v8 dataset.
(TIF)

S6 Fig RV eGenes detected from 49 tissues in the GTEx v8 dataset. A. The relationship between the number of eGenes detected by LRT-q per expressed gene and the sample size of each tissue. B. The number of novel RV eGenes (not detected from CV eGenes analysis of GTEx) identified by each method. In panel B, only tissues with more than one RV eGene detected by any methods are included.
(TIF)

S7 Fig The overlaps of RV eGenes detected by four methods in four GTEx tissues. It includes the RV eGene overlap among LRT-q, SKAT-O, ACAT-O, and VT in (A) Muscle_Skeletal, (B) Skin_Sun_Exposed_Lower_leg, (C) Thyroid, and (D) Whole_Blood.
(TIF)

S8 Fig Changes in p-values of non-novel RV eGenes detected by LRT-q after regressing out effect of common eQTLs from gene expression. We show the changes in p-values in all eight different weighting schemes after regressing out effect of common eQTLs from gene expression within (A) 20kb from TSS in Muscle_Skeletal, (B) 50kb from TSS in Muscle_Skeletal, (C) 100kb from TSS in Muscle_Skeletal, (D) 20kb from TSS in Skin_Sun_Exposed_Lower_leg, (E) 50kb from TSS in Skin_Sun_Exposed_Lower_leg, (F) 100kb from TSS in Skin_Sun_Exposed_Lower_leg, (G) 20kb from TSS in Thyroid, (H) 50kb from TSS in Thyroid, (I) 100kb from TSS in Thyroid, (J) 20kb from TSS in Whole_Blood, (K) 50kb from TSS in Whole_Blood, and (L) 100kb from TSS in Whole_Blood.
(TIF)

S9 Fig Pairwise tissue-sharing matrix of RV eGenes (FDR < 10%) with clustering. It shows the fraction of shared RV eGenes in each pair of tissues. Here we use FDR <10% to increase the number of RV eGenes. Tissues are sorted in an alphabetical order. Only tissues with more than one RV eGenes are included.
(TIF)

S10 Fig Tissue-sharing patterns of RV eGenes and CV eGenes in the GTEx v8 dataset. A. Pairwise tissue-sharing matrix of CV eGenes (FDR < 5%). It shows the fraction of shared CV eGenes in each pair of tissues. Tissues are sorted in alphabetical order. B. The proportion of RV eGenes and CV eGenes shared among different numbers of tissues. Only tissues with at least 100 RV eGenes are considered. Panel B shows the proportion of tissue- specific eGenes that are only detected in one tissue, in 2–4 tissues, and in more than 4 tissues.
(TIF)

S11 Fig Pairwise tissue-sharing matrix of CV eGenes (FDR < 5%) with clustering. It shows the fraction of shared CV eGenes in each pair of tissues. Tissues are sorted by clustering.
(TIF)

S12 Fig The proportion of outliers with rare variants in all outliers. It shows the proportion of outliers carrying rare variants near the corresponding genes in each tissue. We show the mean values as dots and 95% confidence intervals as error bars.
(TIF)

S13 Fig Enrichment of nearby rare variants in outliers defined with different Z-score thresholds. Eight Z-score cutoff values are compared. The text above the data points represents the number of outliers. We show the mean values as dots and 95% confidence intervals as error bars.
(TIF)

S14 Fig Venn diagram showing the differences of LRT-q with fixed 100k permutations and adaptive permutations. It shows the numbers of RV eGenes detected in GTEx Whole Blood with LRT-q using adaptive permutations and fixed 100k permutations, as well as their overlaps.
(TIF)

S1 Table Number of RV eGenes (FDR < 5%) in whole blood detected by four methods using different weights. (XLSX)

S2 Table Number of total RV eGenes (FDR < 5%) detected by four methods in five tissues. (XLSX)

S3 Table Number of novel RV eGenes (FDR < 5%) detected by four methods in five tissues. (XLSX)

S4 Table Number of total RV eGenes (FDR < 5%) detected in whole blood by four methods using different sets of rare variants. (XLSX)

S5 Table Number of novel RV eGenes (FDR < 5%) detected in whole blood by four methods using different sets of rare variants. (XLSX)

S6 Table RV eGenes and genes with p-values < 1e-4 from LRT-q before and after regressing out common eQTLs. (XLSX)

S7 Table RV eGenes and genes with p-values < 1e-4 from SKAT-O before and after regressing out common eQTLs. (XLSX)

S8 Table Summary statistics of 49 GTEx tissues. (XLSX)
