# Supplementary material for: Rare variants regulate expression of nearby individual genes in multiple tissues
Source: PLoS Genet. 2021 Jun 1;17(6):e1009596. doi: 10.1371/journal.pgen.1009596 (PMC8195400; doi:10.1371/journal.pgen.1009596)
Supplement: S1 Text — It describes the mathematical model of the likelihood ratio test used in LRT-q, the derivations of the equations for parameter estimation in the model, and the decision boundary simulation framework. (DOCX) [file pgen.1009596.s001.docx]

# Supplemental Methods

### **Likelihood ratio test**

Based on the likelihood model, we propose a likelihood ratio test for quantitative traits, especially for gene expression. Define $L_{0}$ as the likelihood under the null hypothesis and $L_{1}$ as the likelihood under the alternative hypothesis. In the original LRT method for binary traits, $L_{0}$ and $L_{1}$ are calculated using the likelihood function for the Bernoulli distribution. Here, using the assumption that expression values are normally distributed, we can calculate $L_{0}$ and $L_{1}$ using the likelihood function for the normal distribution. To make sure of the normality of gene expression values, we recommend researchers perform quantile normalization before applying our methods. Based on the mathematical model of the original LRT method for binary traits, we derive the equations for the likelihood ratio test for quantitative traits as following.

According to equation (1) and (2), $L_{0}$ and $L_{1}$ can be computed as

$\begin{matrix} L_{0} & =L(X,Y|V_{0})P(V_{0}) \\ & =\prod_{i=1}^{k} [L(X_{i},Y_{i}|v_{i}=0)(1-c_{i})] \end{matrix}$ (3)

$\begin{matrix} L_{1} & =\sum_{q=1}^{2^{k}-1} L(X,Y|V_{q})P(V_{q}) \\ & =\sum_{q=1}^{2^{k}-1} \prod_{i=1}^{k} \{[L(X_{i},Y_{i}|v_{i}=0) \\ & \cdot(1-c_{i})]^{1-v_{i}^{q}}[L(X_{i},Y_{i}|v_{i}=1)c_{i}]^{v_{i}^{q}}\} \end{matrix}$ (4)

where $V_{0}=\vec{0}$, meaning that there is no causal variant. Hence, $L_{0}$ denotes the likelihood of the observed expression levels under the null hypothesis where there is no causal variant, while $L_{1}$ denotes the likelihood of the observed expression levels under the alternative hypothesis where there is at least one causal variant.

This model differs from the original LRT model^27^ as follows. In the original model, the likelihood was the probabilities of the observed genotypes conditioned on the dichotomous phenotype. Here, we defined the likelihood as the probabilities of the expression levels conditioned on the observed genotypes. There can be many ways to build a generative model of expression levels conditioned on genotypes, where the most common one being the additive model in which the mean of the expression levels shifts by the additive effects of multiple variants. However, using the additive model will make our method computationally infeasible, because we have to apply regression for each $V_{q}$. Therefore, we decided to use an approximated model as in equation (4), where we assume that the same expression levels were generated from multiple distributions corresponding to multiple variants. This is similar to assuming we observed $k$ copies of the same expression levels given $k$ variants. This approximation allows us to decompose the equation to reduce the computational complexity dramatically as follows while providing good power as shown in Results.

The time complexity of calculating $L_{1}$ is $O(2^{k}k)$, which may be too computationally intensive to compute it directly. As described in the original LRT paper, we can calculate $L_{1}$ in linear time using the following decomposition.

$\begin{matrix} L_{0}+L_{1} & =\sum_{q=0}^{2^{k}-1} \prod_{i=1}^{k} A_{i} \\ & =[L(X_{1},Y_{1}|v_{1}=0)(1-c_{1})]\cdot\sum_{q=0}^{2^{k-1}-1} \prod_{i=2}^{k} A_{i} \\ & +[L(X_{1},Y_{1}|v_{1}=1)c_{1}]\cdot\sum_{q=0}^{2^{k-1}-1} \prod_{i=2}^{k} A_{i} \\ & =[L(X_{1},Y_{1}|v_{1}=0)(1-c_{1})+L(X_{1},Y_{1}|v_{1}=1)c_{1}] \\ & \cdot\sum_{q=0}^{2^{k-1}-1} \prod_{i=2}^{k} A_{i} \\ & =\cdots\\ & =\prod_{i=1}^{k} [(1-c_{i})L(X_{i},Y_{i}|v_{i}=0)+c_{i}L(X_{i},Y_{i}|v_{i}=1)] \end{matrix}$ (5)

where

$$A_{i}=[L(X_{i},Y_{i}|v_{i}=0)(1-c_{i})]^{1-v_{i}^{q}}[L(X_{i},Y_{i}|v_{i}=1)c_{i}]^{v_{i}^{q}}$$

Then we can calculate $L_{1}=(L_{0}+L_{1})-L_{0}$ in linear time ($O(k)$), which is much faster.
As $X$ and $Y$ are assumed to be normally distributed with shared variance, equation (3) and (4) can be written as

$\begin{matrix} L_{0}=\prod_{i=1}^{k} \{(1-c_{i})(\frac{1}{2\pi\sigma^{2}})^{\frac{m_{i}+n_{i}}{2}} \\ \cdot exp\{-\frac{1}{2\sigma^{2}}[\sum_{t=1}^{m_{i}} (x_{i}^{t}-\mu)^{2}+\sum_{j=1}^{n_{i}} (y_{i}^{j}-\mu)^{2}]\}\} \end{matrix}$ (6)

$\begin{matrix} L_{0}+L_{1}=\prod_{i=1}^{k} \{(1-c_{i})(\frac{1}{2\pi\sigma^{2}})^{\frac{m_{i}+n_{i}}{2}} \\ \cdot exp\{-\frac{1}{2\sigma^{2}}[\sum_{t=1}^{m_{i}} (x_{i}^{t}-\mu)^{2}+\sum_{j=1}^{n_{i}} (y_{i}^{j}-\mu)^{2}]\} \\ +c_{i}(\frac{1}{2\pi\sigma_{i}^{2}})^{\frac{(m_{i}+n_{i})}{2}} \\ \cdot exp\{-\frac{1}{2\sigma_{i}^{2}}[\sum_{t=1}^{m_{i}} (x_{i}^{t}-\mu_{X_{i}})^{2}+\sum_{j=1}^{n_{i}} (y_{i}^{j}-\mu_{Y_{i}})^{2}]\}\} \end{matrix}$ (7)

where $\mu$ and $\sigma$ are the mean and variance of gene expression levels of all individuals (combining $X$ and $Y$), respectively.

Finally, the statistic of this likelihood ratio test is given by

$\Lambda=\frac{L_{0}}{L_{1}} =\frac{L_{0}}{(L_{0}+L_{1})-L_{0}}$ (8)

To calculate the p-value for this statistic, we use a permutation test because the distribution of the statistic is unknown. We calculate the LRT-q statistic for the observed (unpermuted) data and each permuted data. Then p-value is given as follows.

$p=\frac{\# of (\Lambda_{permuted}\geq\Lambda_{observed})+1}{\# of permutations+1}$ (9)

### **Parameter estimation**

In this model, there are parameters to be estimated: $\mu,\sigma,\mu_{X_{i}},\mu_{Y_{i}},\sigma_{i}$, where$i$ represents the rare variant$i (1\leq i\leq k, k is the total number of rare variants)$. The basic idea of estimating these parameters is to find the values that can maximize $L_{0}$ and $L_{1}$. We derived the maximum likelihood estimators from equation (6) and (7), which is shown below.

$\begin{matrix} \overset{̂}{\mu}=\frac{\sum_{i=1}^{k} (\sum_{t=1}^{m_{i}} x_{i}^{t}+\sum_{j=1}^{n_{i}} y_{i}^{j})}{\sum_{i=1}^{k} (m_{i}+n_{i})} \end{matrix}$ (10)

$\begin{matrix} {\overset{̂}{\sigma}}^{2}=\frac{\sum_{i=1}^{k} [\sum_{t=1}^{m_{i}} (x_{i}^{t}-\mu)^{2}+\sum_{j=1}^{n_{i}} (y_{i}^{j}-\mu)^{2}]}{\sum_{i=1}^{k} (m_{i}+n_{i})} \end{matrix}$ (11)

$\begin{matrix} {\overset{̂}{\mu}}_{X_{i}}=\frac{\sum_{t=1}^{m_{i}} x_{i}^{t}}{m_{i}} \end{matrix}$ (12)

$\begin{matrix} {\overset{̂}{\mu}}_{Y_{i}}=\frac{\sum_{j=1}^{n_{i}} y_{i}^{j}}{n_{i}} \end{matrix}$ (13)

$\begin{matrix} {\overset{̂}{\sigma}}_{i}^{2}=\frac{\sum_{t=1}^{m_{i}} (x_{i}^{t}-{\overset{̂}{\mu}}_{X_{i}})^{2}+\sum_{j=1}^{n_{i}} (y_{i}^{j}-{\overset{̂}{\mu}}_{Y_{i}})^{2}}{m_{i}+n_{i}} \end{matrix}$ (14)

where $\hat{\mu},\hat{\sigma}^{2}, \hat{\mu}_{X_{i}}, \hat{\mu}_{Y_{i}},\hat{\sigma}_{i}^{2}$ represent the estimate of $\mu,\sigma,\mu_{X_{i}},\mu_{Y_{i}},\sigma_{i}$, respectively.

### **Decision boundary simulation**

We generate simulations to visualize decision boundaries of different rare variant methods for finding genes whose rare variants influence gene expression. Following the decision boundary simulation framework in Sul et al.’s work^27^, we design a simulation experiment with 100,000 datasets, each of which has 1,000 individuals. Genotypes and phenotypes are generated according to the power simulations, but we use only two rare variants for a gene in this simulation. Each of the two rare variants has 50% probability of being causal ($c_{i}=0.5$). We set $a=0.9$, that is, the maximum effect size of a causal variant would be $2.97$ in the simulation. In each dataset, to determine the significant relationship between the quantitative trait and each rare variant, we perform a single variant test for each variant using linear regression where t-test is performed with the null hypothesis that the regression coefficient of this rare variant is zero, generating a t-statistic for each of the two rare variants. We plot these t-statistics in 2-D plots where the x-axis is a t-statistic for one rare variant and the y-axis is a t-statistics for the other rare variant. Additionally, we apply LRT-q, VT, CMC, and SKAT-O to both rare variants to identify whether they are collectively associated with the quantitative trait at the significance level $\alpha=0.05$. VT and LRT-q are run with 10,000 permutations.
