# Supplementary figures and images for: Rare variants regulate expression of nearby individual genes in multiple tissues
Source: PLoS Genet. 2021 Jun 1;17(6):e1009596. doi: 10.1371/journal.pgen.1009596 (PMC8195400; doi:10.1371/journal.pgen.1009596)

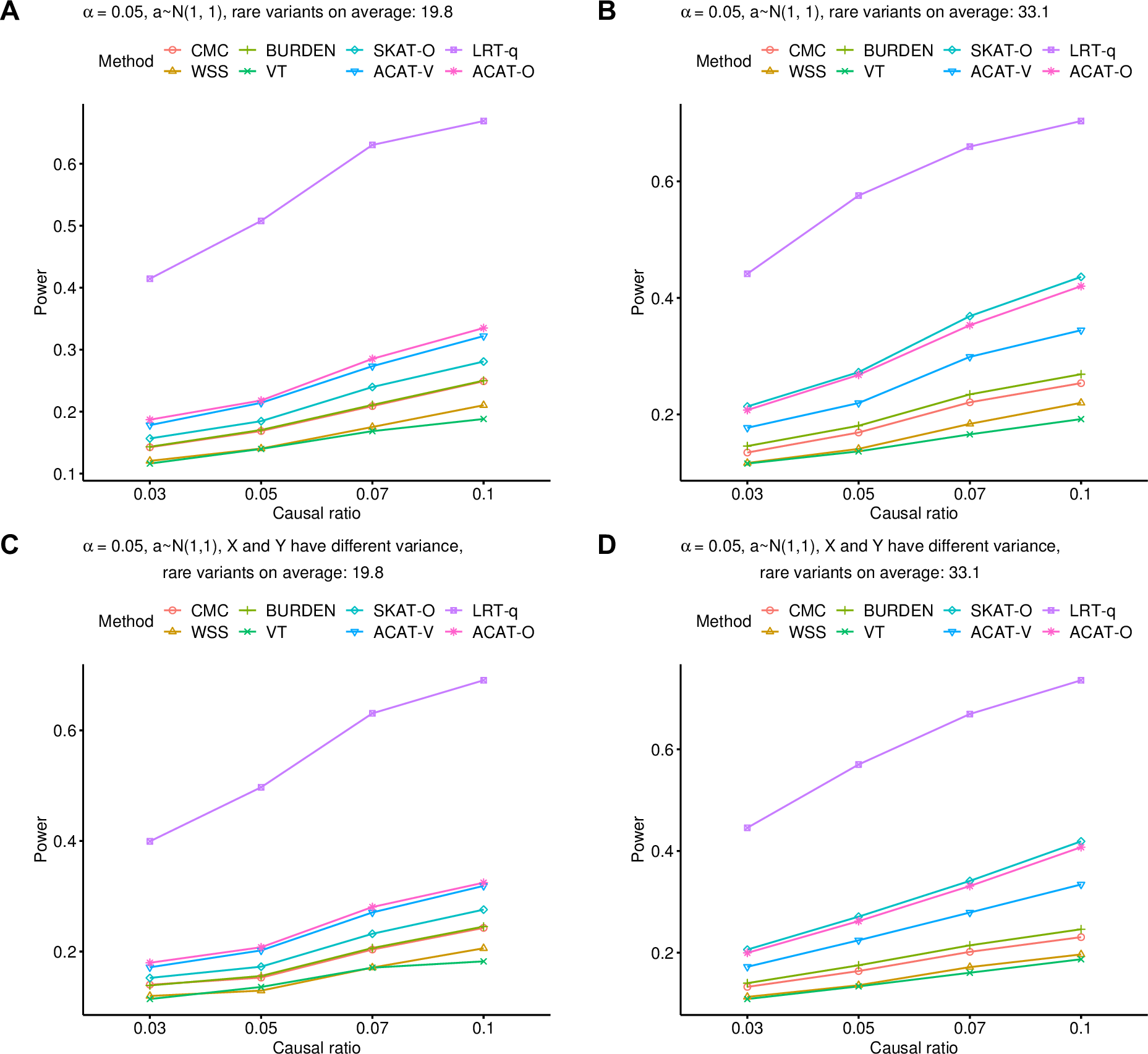

Supplement: S1 Fig — We sample a from a normal distribution N(1,1) and simulate genotypes with (A) 19.8 rare variants on average and (B) 33.1 rare variants on average. We also simulate X and Y to have explicitly different variances with a sampled from a normal distribution N(1,1), and then perform association tests on the simulated genotypes with (C) 19.8 rare variants on average and (D) 33.1 rare variants on average. (TIF) [file pgen.1009596.s002.tif]

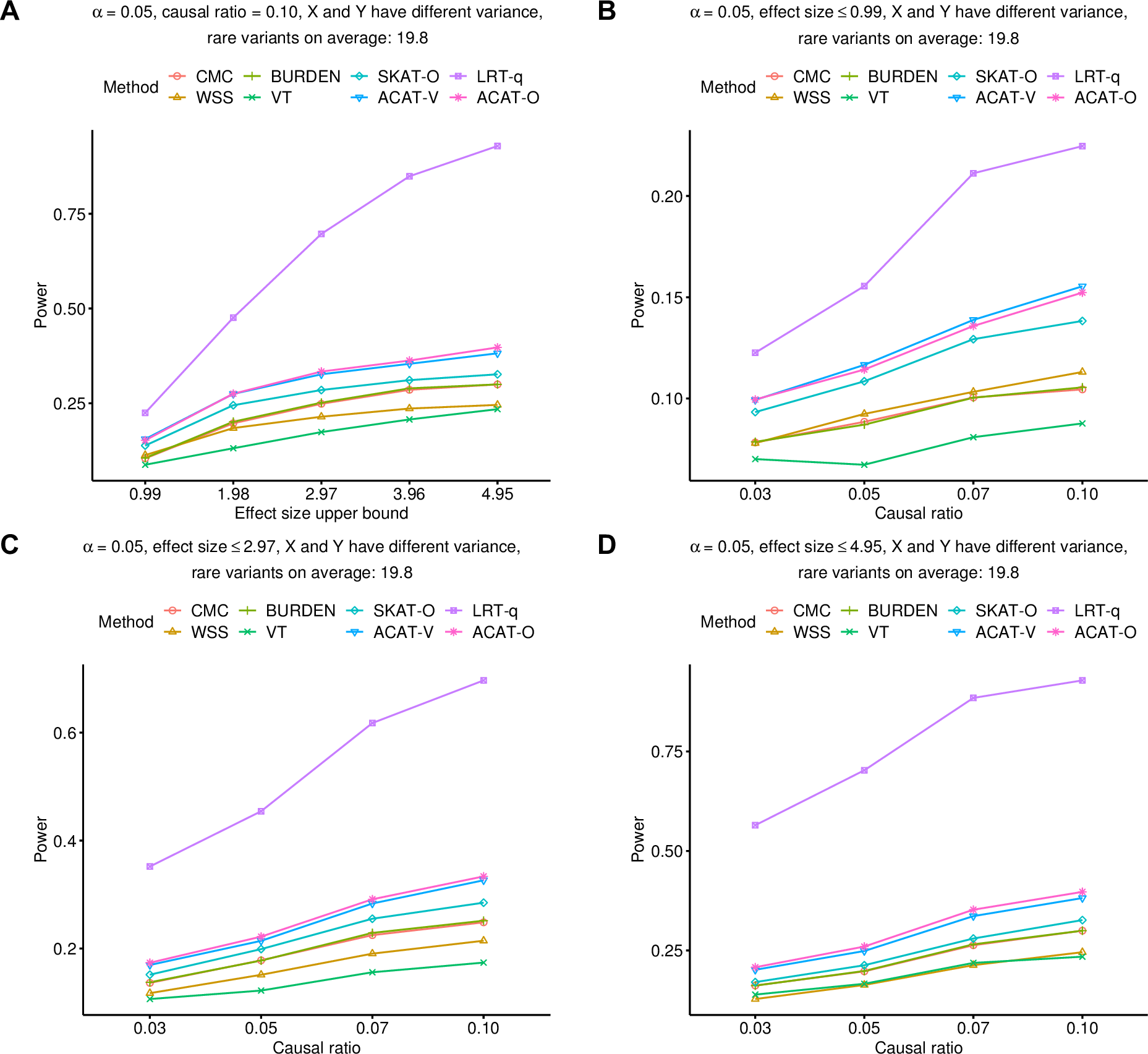

Supplement: S2 Fig — A. for different effect sizes and fixed causal ratio (10%), and for fixed effect sizes (B. ≤ 0.99, C. ≤ 2.97, D. ≤ 4.95) and various causal ratios. Significance level α = 0.05. (TIF) [file pgen.1009596.s003.tif]

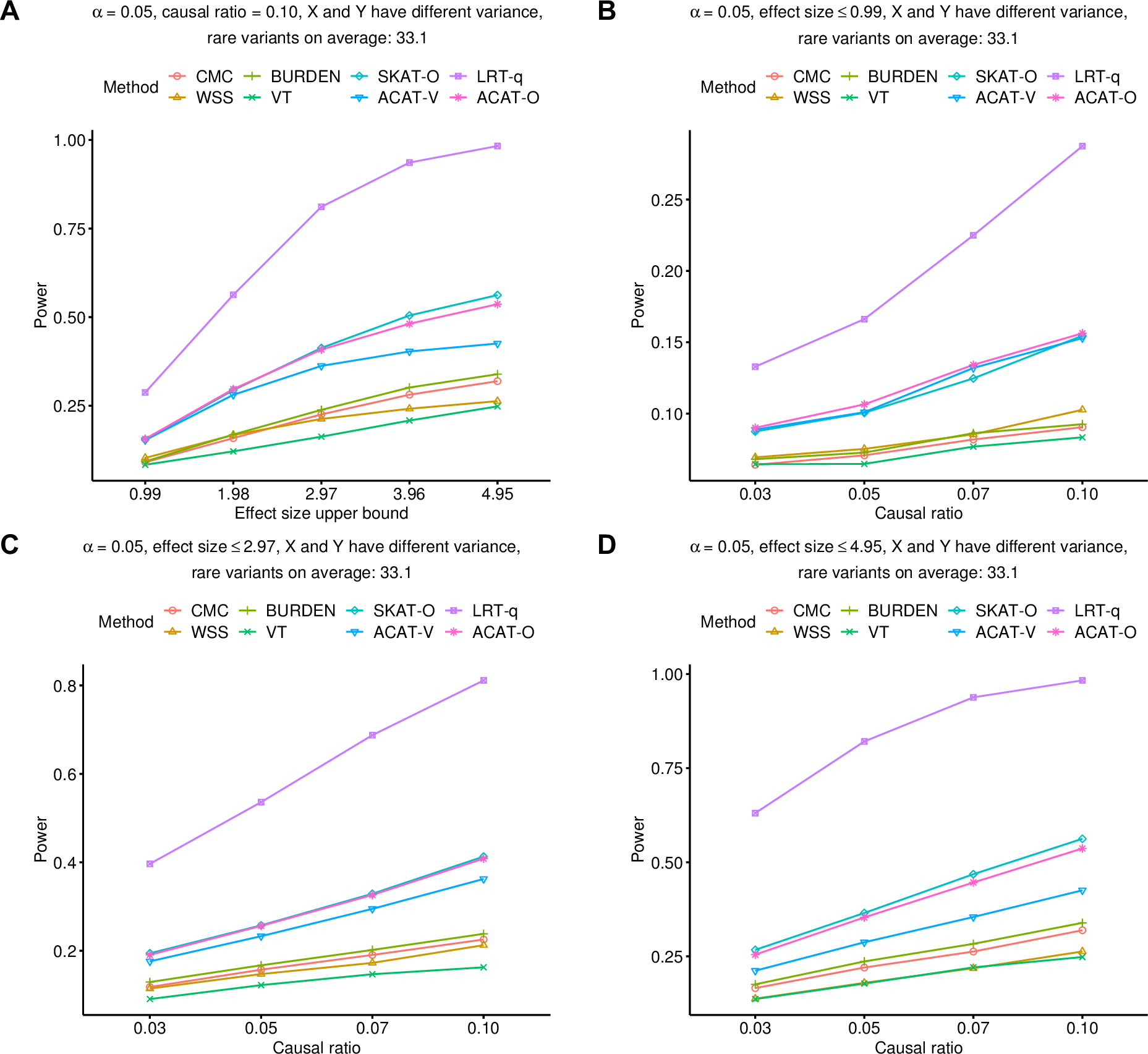

Supplement: S3 Fig — A. for different effect sizes and fixed causal ratio (10%), and for fixed effect sizes (B. ≤ 0.99, C. ≤ 2.97, D. ≤ 4.95) and various causal ratios. Significance level α = 0.05. (TIF) [file pgen.1009596.s004.tif]

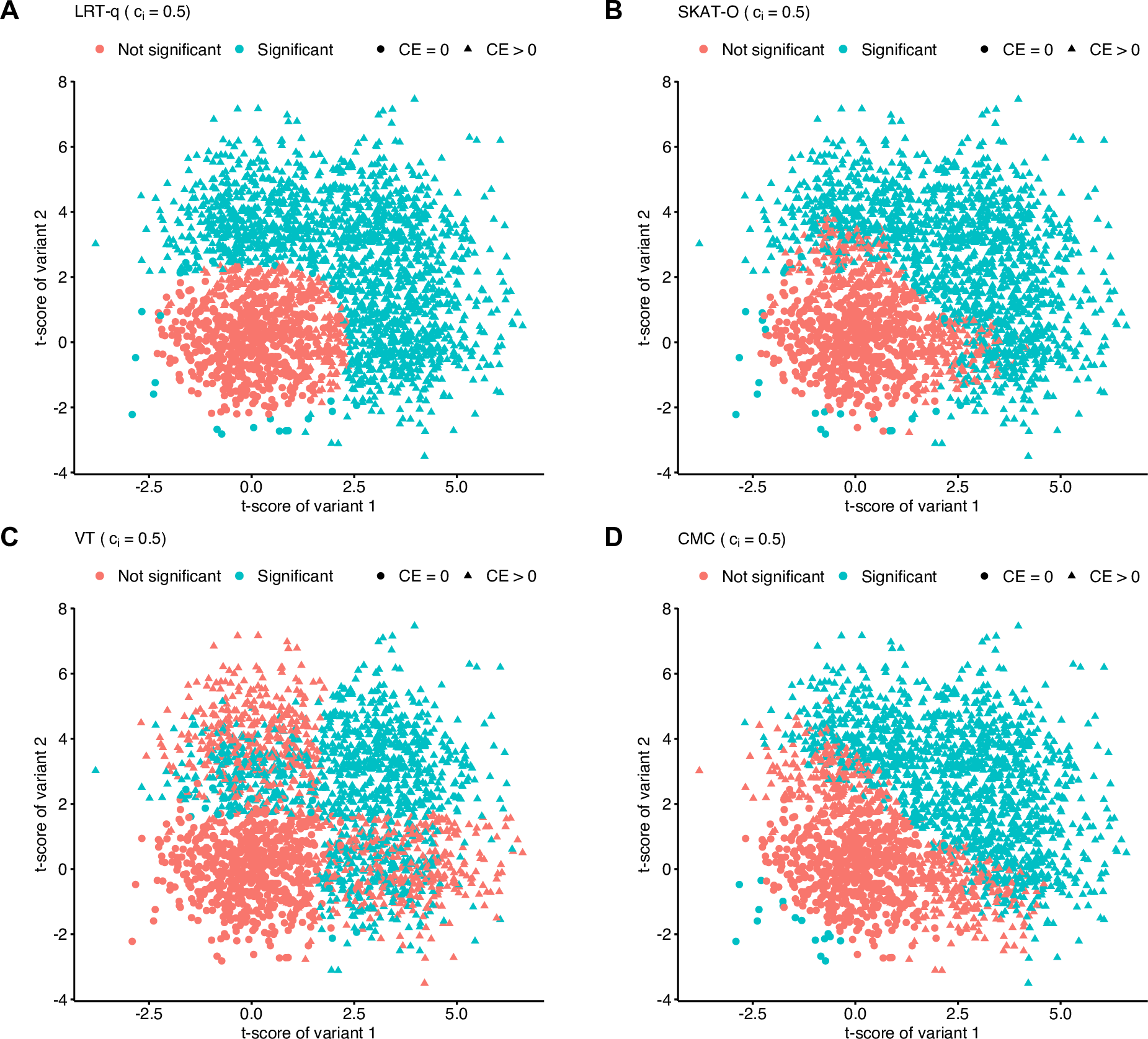

Supplement: S4 Fig — It shows the decision boundary of A. LRT-q, B. SKAT-O, C. VT, D. CMC at ci = 0.5. ci represents the probability of a rare variant being causal. Each data point is labeled as significant if a significant association is identified, or not significant otherwise. "CE = 0": there are no effects of causal variants in the dataset because there are no causal variants. "CE > 0": there are effects of causal variants in the dataset because there are at least one causal variants. We randomly sample 2,500 data points to show to avoid overplotting. (TIF) [file pgen.1009596.s005.tif]

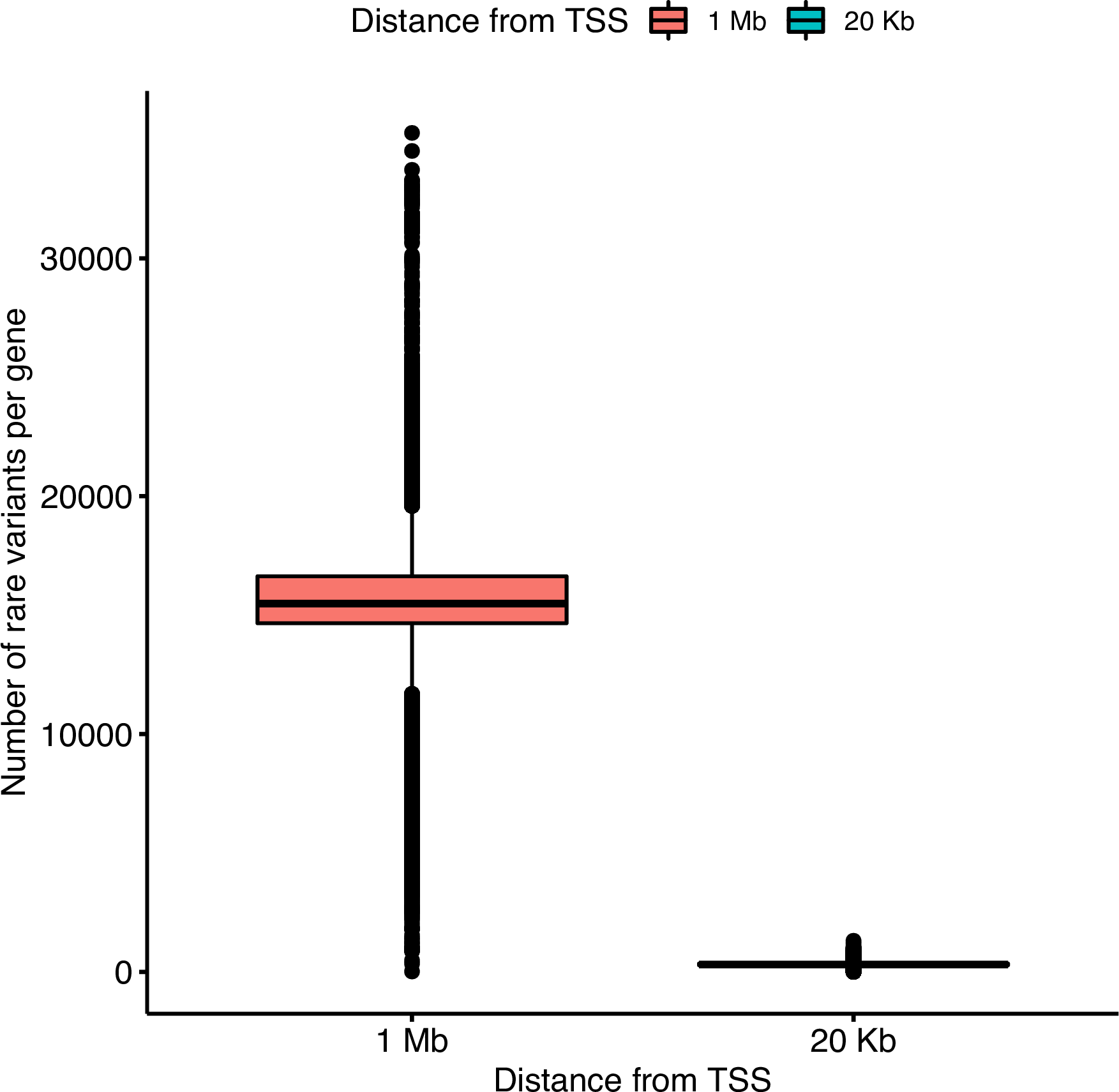

Supplement: S5 Fig — It shows the distribution of the number of rare variants in a window of 1 Mb (median: 15,482) and 20 Kb (median: 311) around TSS across all genes in the GTEx v8 dataset. (TIF) [file pgen.1009596.s006.tif]

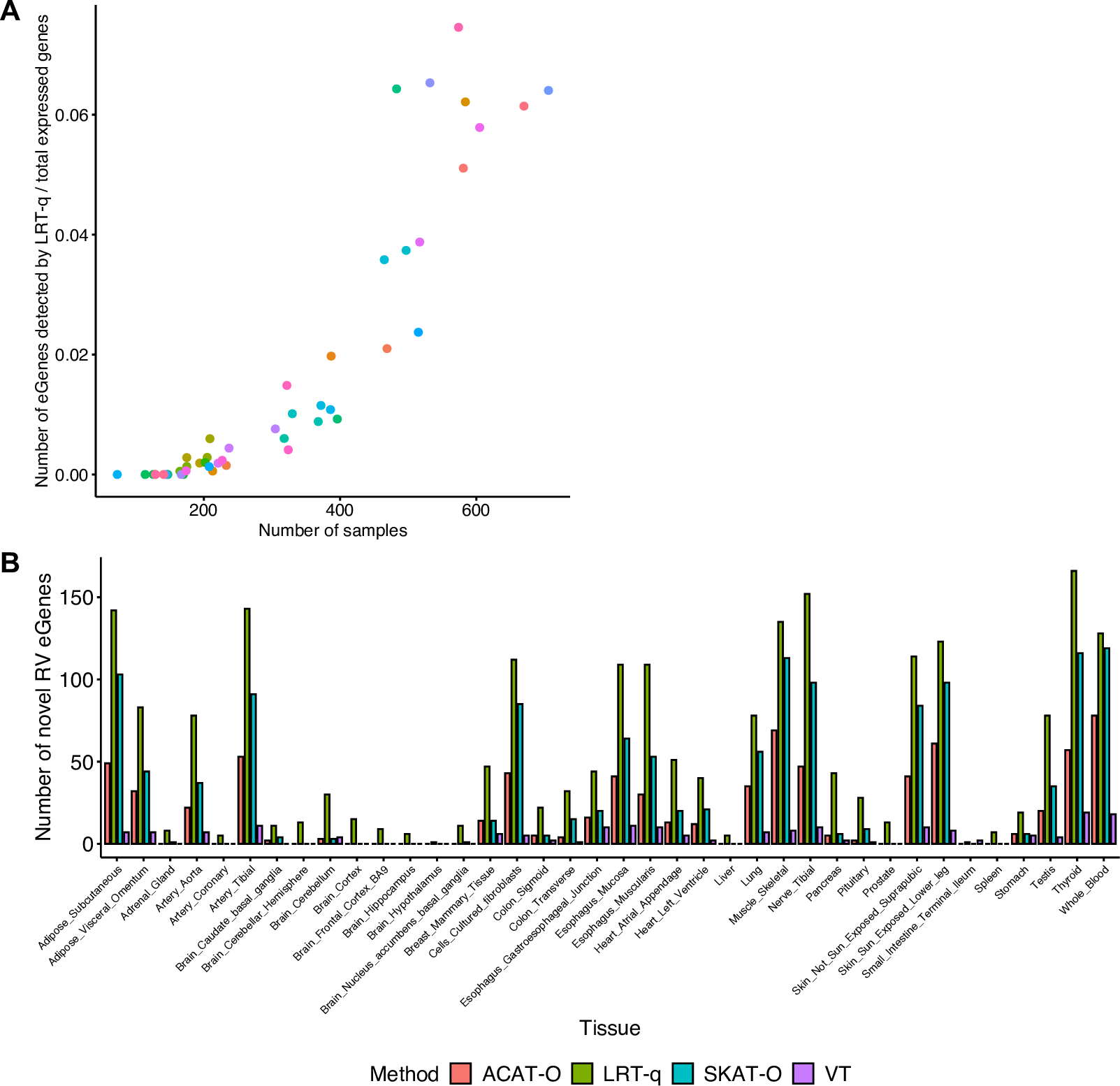

Supplement: S6 Fig — A. The relationship between the number of eGenes detected by LRT-q per expressed gene and the sample size of each tissue. B. The number of novel RV eGenes (not detected from CV eGenes analysis of GTEx) identified by each method. In panel B, only tissues with more than one RV eGene detected by any methods are included. (TIF) [file pgen.1009596.s007.tif]

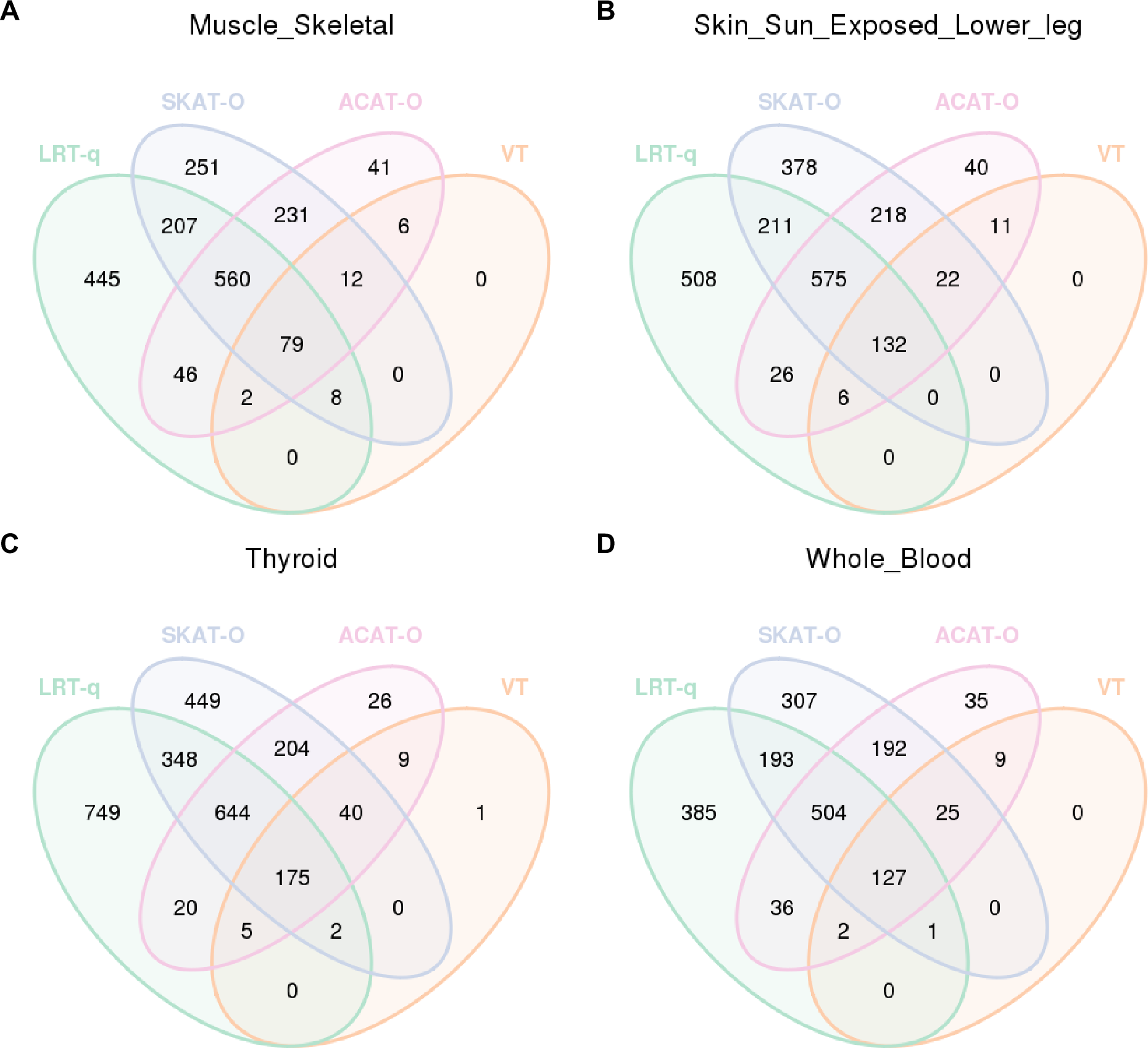

Supplement: S7 Fig — It includes the RV eGene overlap among LRT-q, SKAT-O, ACAT-O, and VT in (A) Muscle_Skeletal, (B) Skin_Sun_Exposed_Lower_leg, (C) Thyroid, and (D) Whole_Blood. (TIF) [file pgen.1009596.s008.tif]

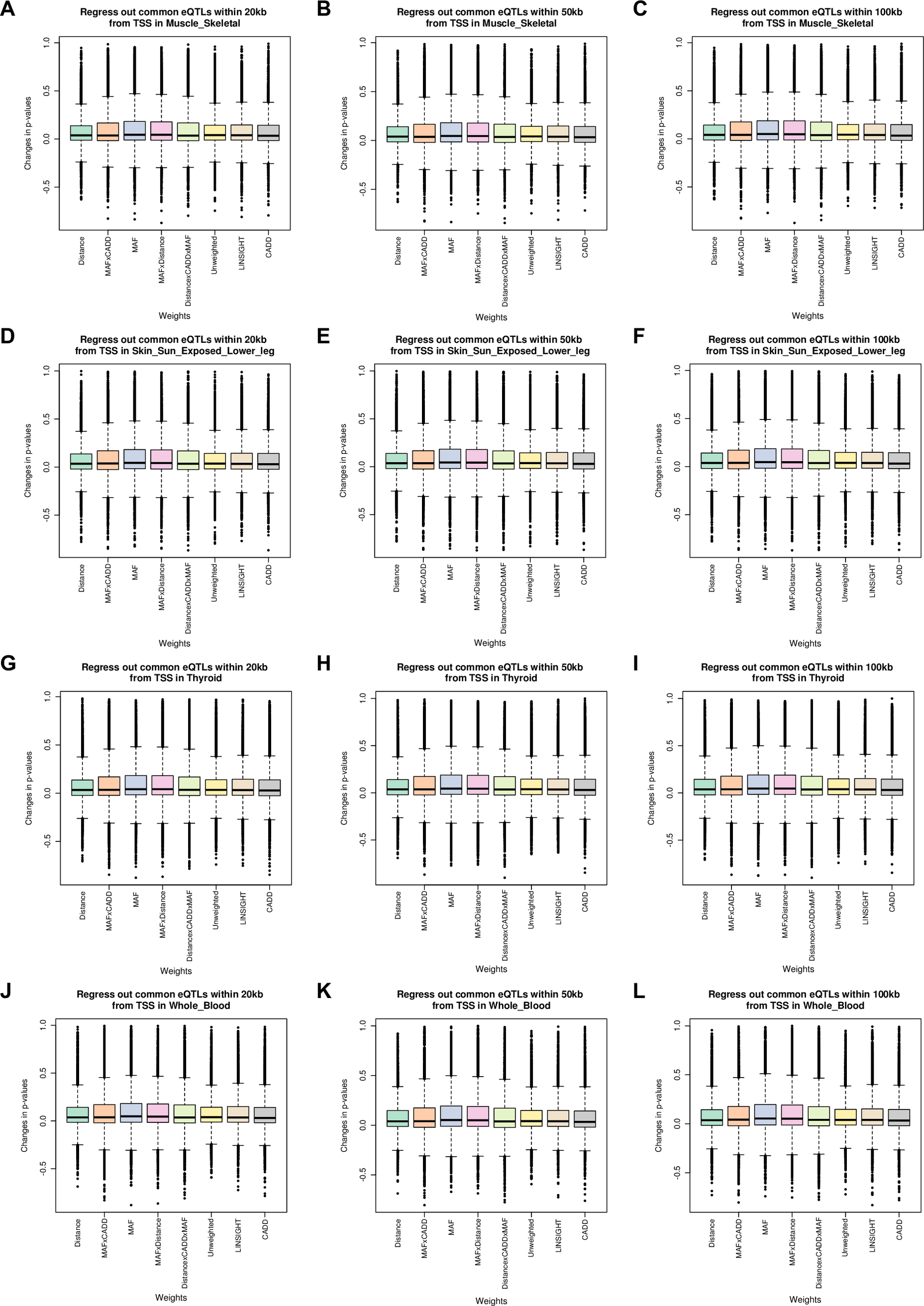

Supplement: S8 Fig — We show the changes in p-values in all eight different weighting schemes after regressing out effect of common eQTLs from gene expression within (A) 20kb from TSS in Muscle_Skeletal, (B) 50kb from TSS in Muscle_Skeletal, (C) 100kb from TSS in Muscle_Skeletal, (D) 20kb from TSS in Skin_Sun_Exposed_Lower_leg, (E) 50kb from TSS in Skin_Sun_Exposed_Lower_leg, (F) 100kb from TSS in Skin_Sun_Exposed_Lower_leg, (G) 20kb from TSS in Thyroid, (H) 50kb from TSS in Thyroid, (I) 100kb from TSS in Thyroid, (J) 20kb from TSS in Whole_Blood, (K) 50kb from TSS in Whole_Blood, and (L) 100kb from TSS in Whole_Blood. (TIF) [file pgen.1009596.s009.tif]

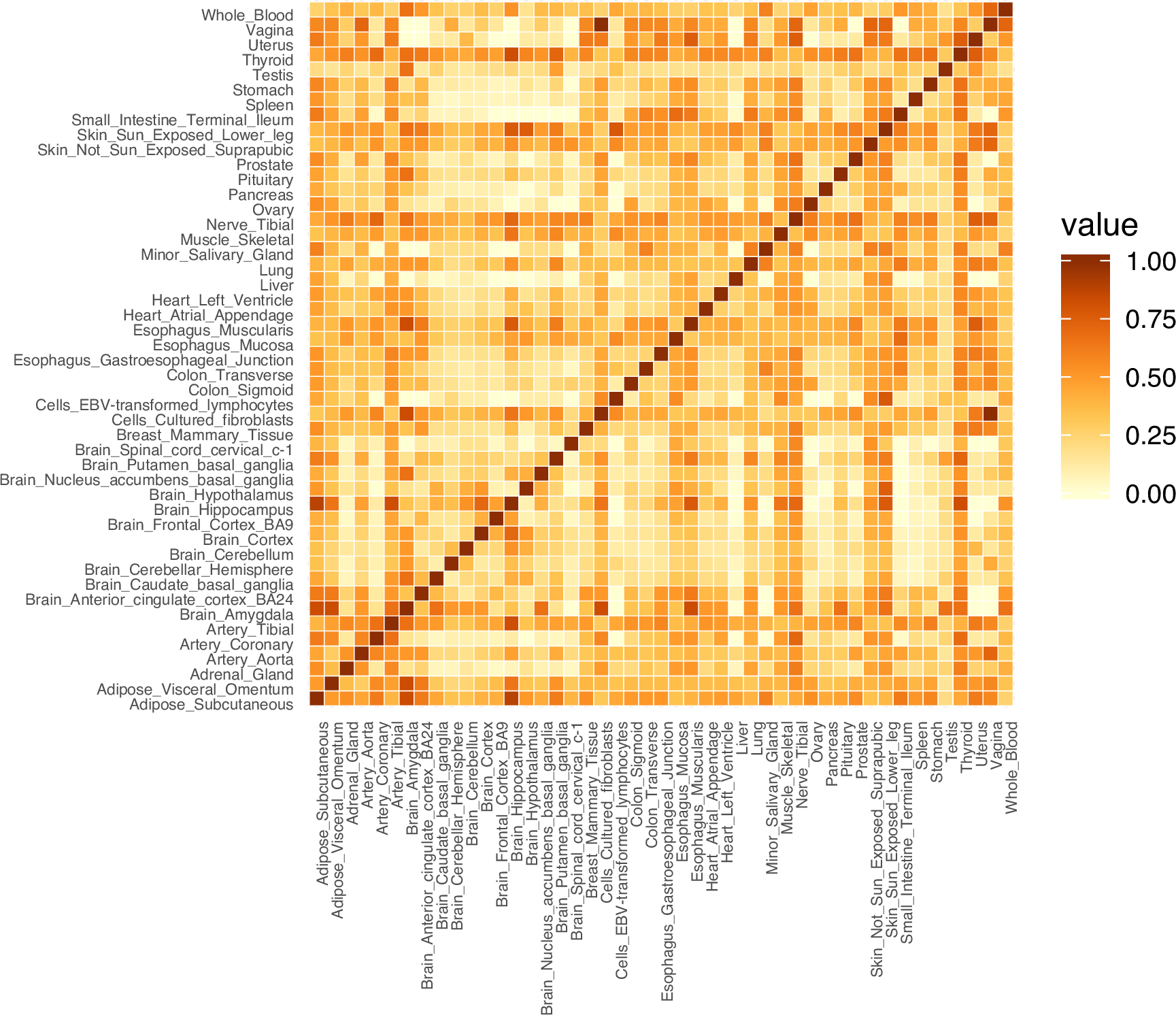

Supplement: S9 Fig — It shows the fraction of shared RV eGenes in each pair of tissues. Here we use FDR <10% to increase the number of RV eGenes. Tissues are sorted in an alphabetical order. Only tissues with more than one RV eGenes are included. (TIF) [file pgen.1009596.s010.tif]

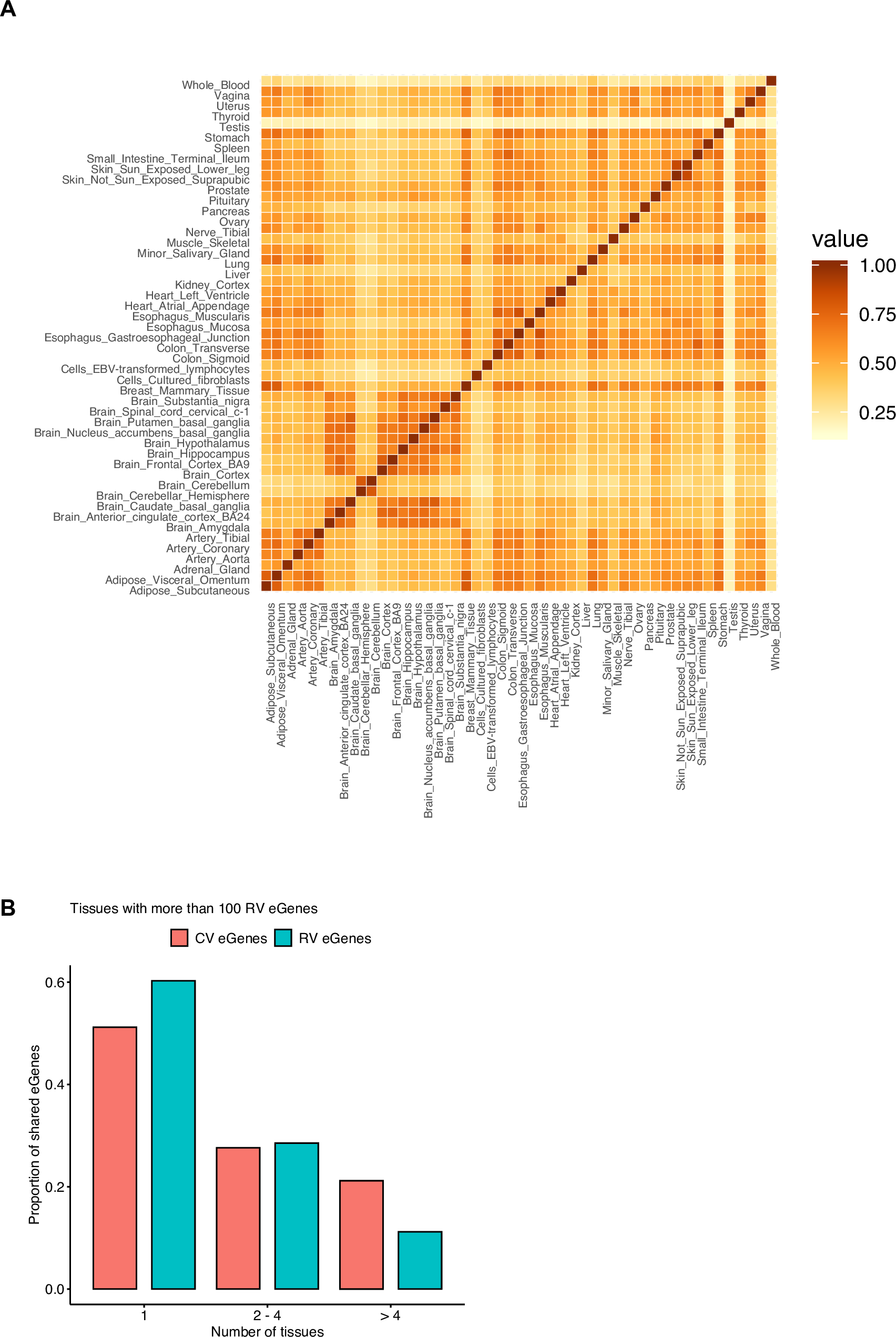

Supplement: S10 Fig — A. Pairwise tissue-sharing matrix of CV eGenes (FDR < 5%). It shows the fraction of shared CV eGenes in each pair of tissues. Tissues are sorted in alphabetical order. B. The proportion of RV eGenes and CV eGenes shared among different numbers of tissues. Only tissues with at least 100 RV eGenes are considered. Panel B shows the proportion of tissue- specific eGenes that are only detected in one tissue, in 2–4 tissues, and in more than 4 tissues. (TIF) [file pgen.1009596.s011.tif]

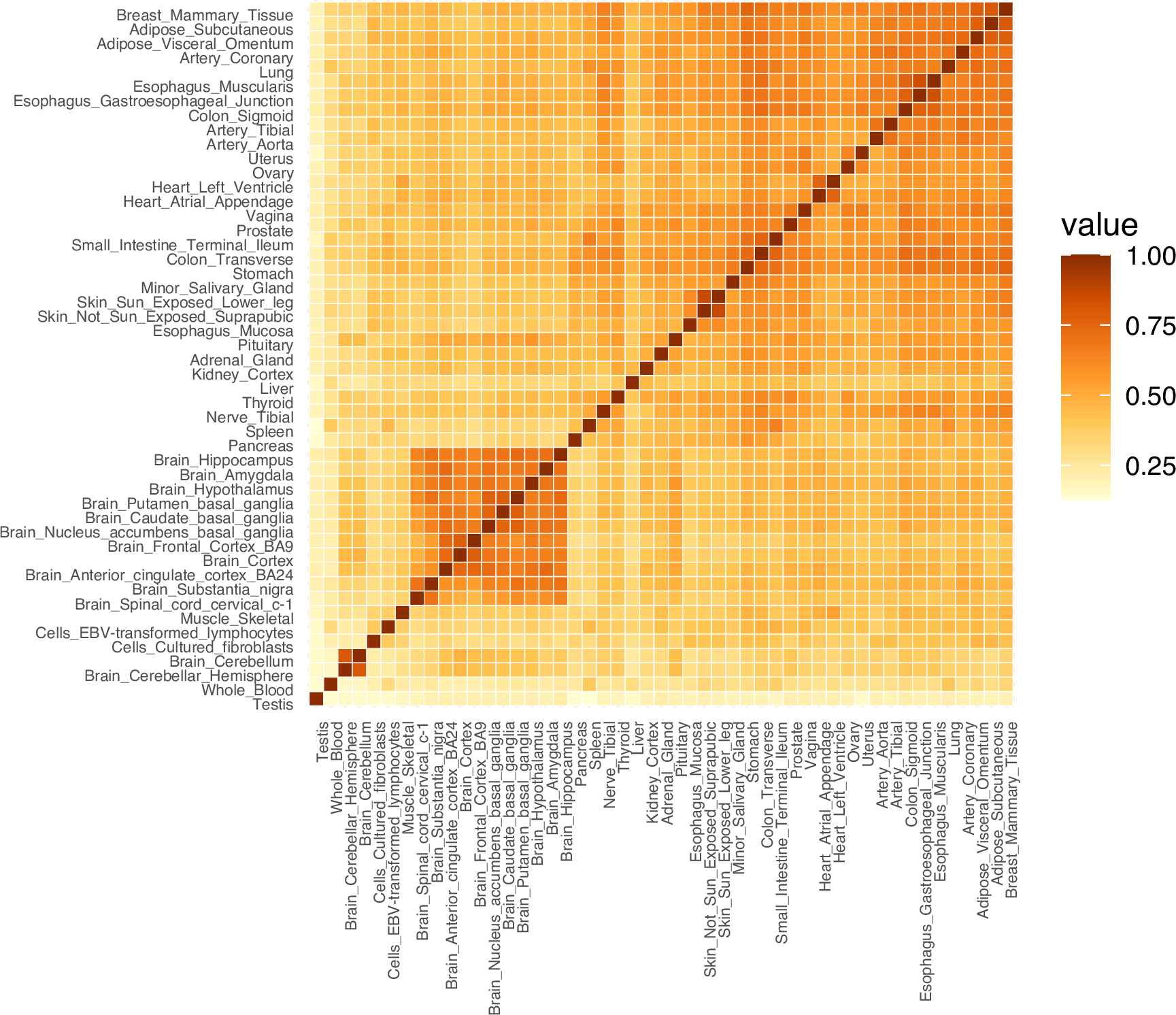

Supplement: S11 Fig — It shows the fraction of shared CV eGenes in each pair of tissues. Tissues are sorted by clustering. (TIF) [file pgen.1009596.s012.tif]

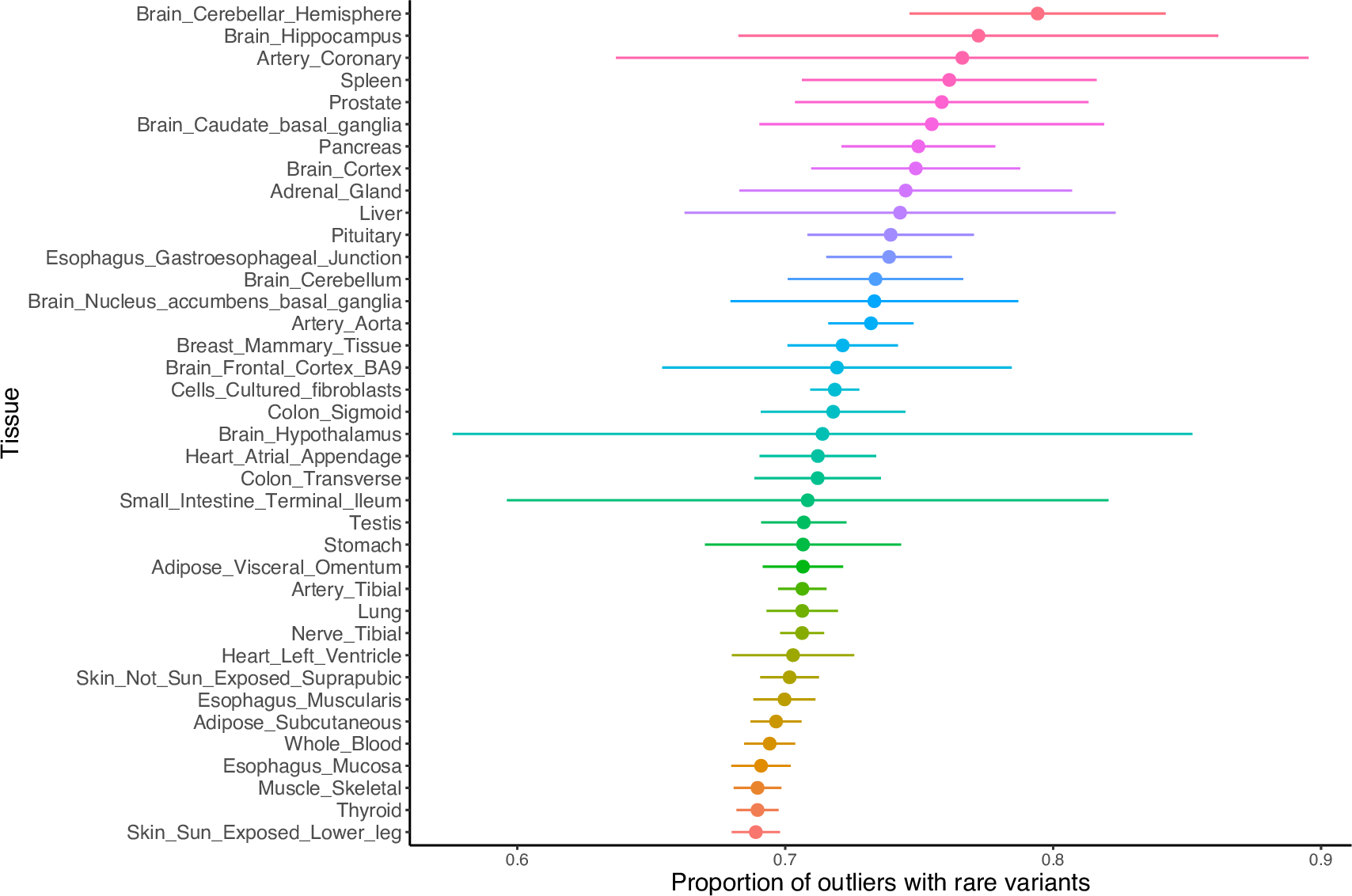

Supplement: S12 Fig — It shows the proportion of outliers carrying rare variants near the corresponding genes in each tissue. We show the mean values as dots and 95% confidence intervals as error bars. (TIF) [file pgen.1009596.s013.tif]

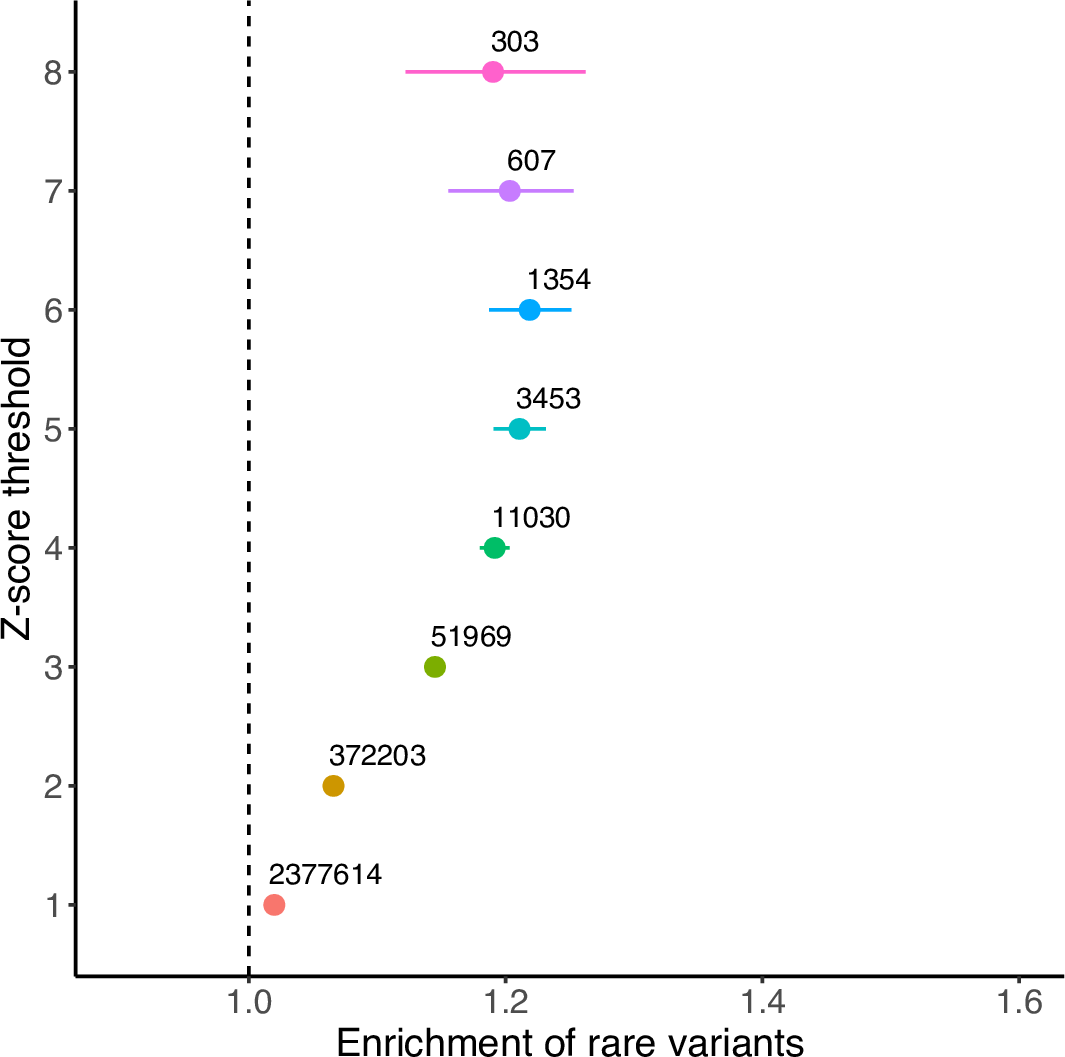

Supplement: S13 Fig — Eight Z-score cutoff values are compared. The text above the data points represents the number of outliers. We show the mean values as dots and 95% confidence intervals as error bars. (TIF) [file pgen.1009596.s014.tif]

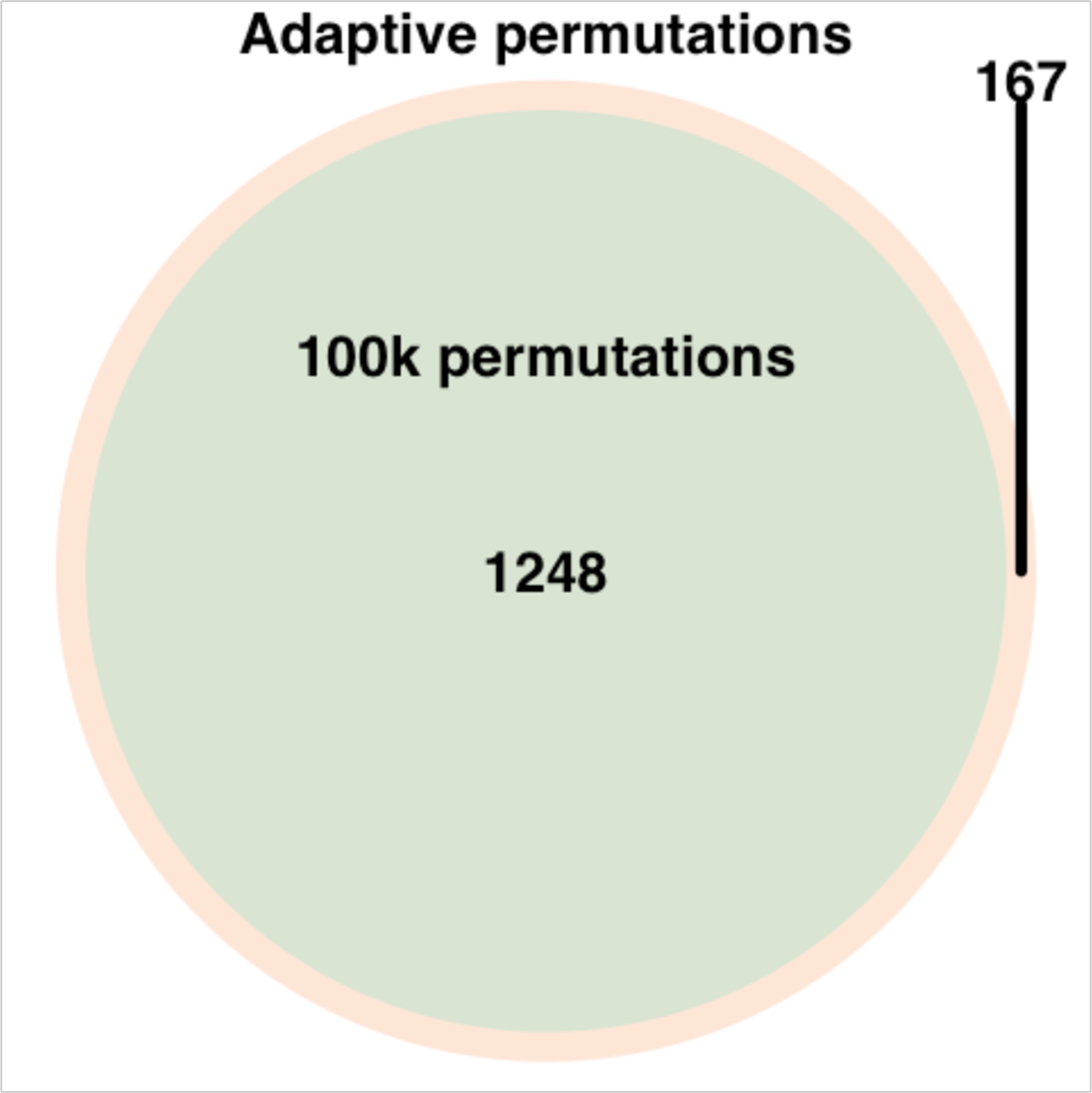

Supplement: S14 Fig — It shows the numbers of RV eGenes detected in GTEx Whole Blood with LRT-q using adaptive permutations and fixed 100k permutations, as well as their overlaps. (TIF) [file pgen.1009596.s015.tif]
